# Atomic-Resolution Structure of the Protein Encoded by Gene V of fd Bacteriophage in Complex with Viral ssDNA Determined by Magic-Angle Spinning Solid-State NMR

**DOI:** 10.1021/jacs.2c09957

**Published:** 2022-12-21

**Authors:** Yoav Shamir, Amir Goldbourt

**Affiliations:** School of Chemistry, Tel Aviv University, Tel Aviv 6997801, Israel

## Abstract

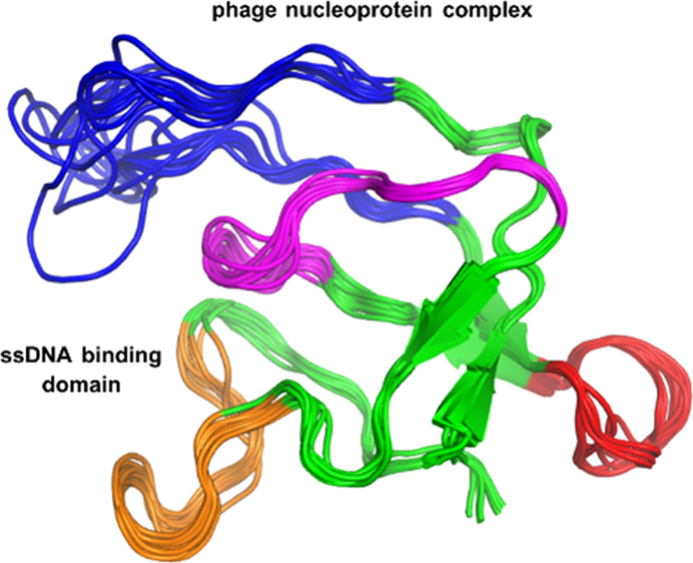

F-specific filamentous phages, elongated particles with circular single-stranded DNA encased in a symmetric protein capsid, undergo an intermediate step, where thousands of homodimers of a non-structural protein, gVp, bind to newly synthesized strands of DNA, preventing further DNA replication and preparing the circular genome in an elongated conformation for assembly of a new virion structure at the membrane. While the structure of the free homodimer is known, the ssDNA-bound conformation has yet to be determined. We report an atomic-resolution structure of the gVp monomer bound to ssDNA of fd phage in the nucleoprotein complex elucidated via magic-angle spinning solid-state NMR. The model presents significant conformational changes with respect to the free form. These modifications facilitate the binding mechanism and possibly promote cooperative binding in the assembly of the gVp–ssDNA complex.

## Introduction

Bacteriophages, the most abundant entities on earth, are viruses that infect bacteria. Filamentous bacteriophages of the genus *Inovirus* are a family of viruses that predominantly infect Gram-negative bacteria.^1,2^ Most of the current biological understanding of filamentous phages, including the phage mechanisms of infection, replication, and assembly, comes from extensive research conducted on a group of three closely related phages, known as F-specific filamentous phages (Ff phages).^3,4^ Ff phages all infect *Escherichia coli* cells bearing F-pili organelles. The members of the Ff group—phages M13, fd, and f1—exhibit 98% DNA sequence identity, and therefore have been studied interchangeably.^2,3,5^ Uniquely among other bacteriophages, infection by filamentous phages does not cause lysis of the host cell^6^ and the host bacterial cells continue to grow and divide as new virion structures are produced within their cytoplasm, with about one-half to three-quarters the growth rate of uninfected cells.^7,8^ First isolated from sewage systems in the early 1960s, Ff phages have since become prominent model systems, used widely in molecular biology research.^9−11^

Ff phage particles have an elongated structure, approximately a micron in length and 6–7 nm in diameter.^12,13^ The virion genome is structured as circular single-stranded DNA, encased in a symmetric protein capsid. Within the capsid, the ssDNA forms an anti-parallel two-stranded helix, structurally similar to double-stranded DNA, but it does not exhibit base-pair complementarity. Most of the capsid is composed of approximately 2700 copies of a 50-residue-long, mostly helical single-coat protein, gVIIIp, known as the major coat protein.^6^ Four minor coat proteins, with only a few copies of each, cap both ends of the virion (gIIIp and gVIp cap one end, while gVIIp and gIXp cap the other end).^6^ The wild-type Ff phage genome comprises around 6400 nucleotides, and it contains nine genes, encoding for a total of 11 proteins.^4,14^

The initial stage of the Ff phage life cycle is the infection of the *Escherichia coli* cell via attachment of gIIIp to the tip of the F-pilus, a helical surface filament that extends from the bacterial surface, containing stoichiometric amounts of proteins and phospholipids.^15,16^ The pilus retracts toward the cell envelope, leading to the opening of the virion structure^17^ and entry of the viral ssDNA into the cytoplasm of the host cell.

Next, the phage genome undergoes episomal replication within the host cell. The positive strand of the genome is used as a template to synthesize a negative strand, and together, both strands form a double-stranded phage genome, known as the replicative form (RF).^3^ In the early stage after infection, RF is used for transcription of phage mRNA, followed by translation and expression of viral proteins.^18^ RF undergoes the rolling circle replication mechanism, resulting in the generation of new positive strands of the phage genome, which are once again utilized for negative-strand synthesis. Once the expression level of a certain phage protein, gVp, has reached a critical threshold within the cytoplasm of the host, thousands of homodimers of gVp cooperatively bind to positive strands of phage ssDNA, coating nearly the entire two-stranded helix and forming a superhelical, left-handed nucleoprotein complex,^19^ known as the premature virion. By disabling synthesis of the negative strand and subsequent generation of RF, further viral genome replication is prevented.^18^ Formation of this nucleoprotein complex also packages the circular ssDNA in an elongated form, making the strand geometrically viable for assembly of the mature virion structure.^1^ Once the intracellular complex reaches the cell membrane, a secretion-assembly process is initiated when an exposed hairpin loop at one end of the premature virion, serving as a packaging signal, interacts with the inner membrane and with phage proteins.^2,4^ Next, in a process that has yet to be characterized in detail, the gVp dimers are stripped from the strand and replaced by the coat proteins, forming the newly assembled mature infectious virion, which is then extruded from the cell.

The binding of gVp dimers to ssDNA is highly cooperative^20^ and is non-sequence-specific.^21^ Depending on experimental conditions, several different binding modes have been described, with various stoichiometric ratios in the range of three to five nucleotides per monomer.^5^ Under physiological in vitro conditions, the predominant stoichiometry reported is of four nucleotides per monomer.^18^

**Figure 1:**
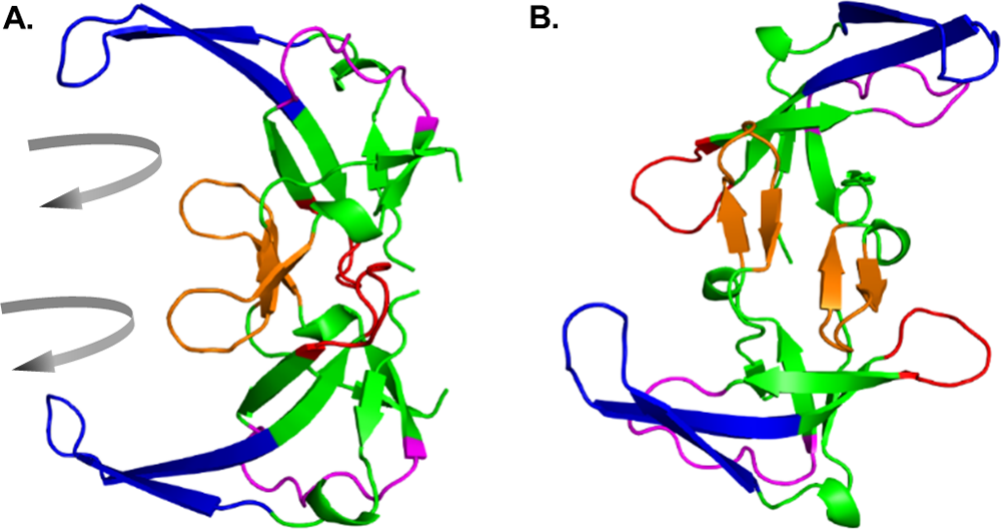
X-ray structure of the free gVp homodimer (reproduced via PyMOL according to PDB ID: 1VQB(22)). (A) Dimer structure with the four main loops colored (DNA binding loop—blue, dyad loop—orange, complex loop—red, core loop—magenta). The two gray arrows represent the hypothesized locations of the two anti-parallel ssDNA strands, each thought to be bound to the protein in a concave cleft formed between the ssDNA binding loop of one monomer and the dyad loop of the second monomer. (B) 90^°^ rotation of the dimer structure around the vertical axis. The DNA strands are omitted here.

Two high-quality models for the structure of free gVp were elucidated, one via X-ray crystallography (Figure 1) for gVp of f1 phage^22^ (PDB ID: 1VQB) and another via solution NMR^23,24^ (PDB ID: 2GVB) for a Y41H mutant of M13 phage gVp, which was found to increase protein solubility.^23^ Both models are nearly identical in secondary and tertiary structures (the backbone root-mean-square deviation (RMSD) of the monomer, excluding the DNA binding loop, is 1.5 Å; for the dimer, it is 1.9 Å^24^) and agree with biochemical and biophysical data previously collected on the system.^18,24^ They differ in the location of the N-terminus with respect to the core, but the main difference involves the orientation of the DNA binding loop with respect to the core. This results in a solution NMR monomer that is slightly more globular than the X-ray structure, and since the former does not seem to have enough vacant volume available in the putative binding cleft, it has previously been suggested that this loop would be required to assume a conformation more similar to that of the X-ray structure for ssDNA binding to take place.^24^

The gVp monomer is 87 residues long. The predominant, stable form in solution, across a range of pH, temperature, protein concentrations, and salt concentrations, is that of a symmetric homodimer, with a total molecular mass of 19.4 kDa.^18^ The homodimer structure in crystals of gVp (Figure 1) is mostly stabilized by inter-monomer hydrophobic interactions.^25,26^ The secondary structure of each monomer includes eight beta-strands and two 3_10_ helices. Five of the beta strands form a right-handed twisted, anti-parallel, distorted beta sheet structure, known as a beta-barrel.^22^ Interestingly, a similar folding motif termed OB (oligonucleotide/oligosaccharide binding) fold, based on a five-stranded beta-barrel, was identified in several oligonucleotide and oligosaccharide binding proteins.^27^ The barrel forms the hydrophobic core of the monomer, from which four main loops protrude.^18^ The first is the complex loop (residues 36–43), which is thought to be involved in dimer–dimer interactions related to the assembly of the nucleoprotein complex.^25^ The second is the dyad loop (residues 68–78). The dyad loop of one monomer is in spatial proximity to that of the other monomer; therefore, they both comprise most of the inter-monomer contact surface of the homodimer.^28^ A third loop region, previously referred to as a “broad connecting loop”^28^ (residues 49–59 connecting the fourth and fifth beta strands, both of which participate in the beta-barrel motif), was reported to be in the vicinity of the viral genome in the complex.^18^ Here, it will be termed the “core loop”. The fourth is the DNA-binding loop (residues 13–31), which was shown to be involved in the binding of the viral genome. Several findings point to the involvement of both the DNA-binding loop and the dyad loop in binding to ssDNA. First, the twofold symmetry axis relating both monomers gives rise to two concave clefts in the dimer structure, each formed by the DNA-binding loop of one monomer and the tip of the dyad loop of the other monomer.^28,29^ These clefts can accommodate the two anti-parallel strands of ssDNA in the complex. Second, NMR studies have implicated residues in both loops as being in spatial proximity to bound nucleotides.^29^ Third, both loops include several amide protons that have been shown to exhibit fast exchange with the solvent, which can indicate that the orientations of both loops with respect to the hydrophobic core are flexible.^18,26,30^ Fourth, the calculated electrostatic potential at the surface of the putative binding domain, between the two loops, is highly positive, while the opposite side of the dimer, located at the outer surface of the nucleoprotein complex, exhibits nearly neutral surface charge.^31^ Such an asymmetric charge distribution can facilitate binding to ssDNA as well as the formation of the symmetric nucleoprotein complex. The latter argument is further corroborated by NMR and fluorescence studies that have indicated that the binding of ssDNA by gVp dimers is mostly facilitated by electrostatic interactions between positively charged side-chain residues of the putative binding regions of the protein and the negatively charged sugar-phosphate ssDNA backbone.^18,32^

The structure of the superhelical gVp–ssDNA nucleoprotein complex has yet to be determined in atomic resolution. No X-ray diffraction structure was reported, and several attempts have been made to computationally calculate a plausible model for the complex structure using global parameters collected empirically on the system. These models utilize the X-ray structure of the free form as a basis for the model, but as of yet, none of the suggested models were proven to be both accurate and in full agreement with available data regarding the complex.^25,31,33^

Magic-angle spinning solid-state NMR (MAS ssNMR) is advantageous for probing systems of nucleotide-bound proteins, since linewidths are not dependent on the particle mass, long-range crystallinity is not required, as well as the ability to sensitively detect conformational changes, as even minor changes in the fold result in significant changes to the resonance positions.^34^ In work previously reported by our lab, the isotropic chemical shifts of both free gVp and ssDNA-bound gVp have been assigned.^35,36^ Quantification and analysis of chemical shift perturbations (CSPs) led to the conclusion that gVp undergoes significant structural changes upon binding, and regions expected to undergo the most extensive structural modifications—including the DNA-binding loop, the core loop, and the C-terminus—were identified.^36^ MAS ssNMR is also useful for protein structure elucidation based on acquisition of internuclear distance-dependent interactions and prediction of backbone torsion angles based on chemical shifts,^37^ as well as acquisition of additional structural restraints.^38,39^ Given a sufficient amount of restraints distributed along different regions of the sequence, a carefully designed calculation process can lead to a converged, viable structural model. Several protein structures have previously been solved via MAS ssNMR, and beyond crystalline monomers, more complex systems include the homodimeric Crh protein,^40^ CAP-Gly bound to microtubules,^41^*α*-synuclein^42^ and additional amyloid aggregates,^43^ and the Anabaena sensory rhodopsin membrane protein in lipid bilayers.^44^

In this research, we describe our calculated, atomic-resolution model for the structure of the gVp monomer in complex with ssDNA of fd phage. We compare the structure to that of free gVp and discuss the biological significance of our findings, which provide insight into the binding process of gVp to Ff phage ssDNA.

## Results and Discussion

### Generation of NMR Distance Restraints

Chemical shift assignments of the ssDNA-bound form of gVp were reported previously^36^ and are available at the Biological Magnetic Resonance Bank (BMRB accession id: 51391). In order to elucidate information on internuclear distances from these assignments, we acquired several two-dimensional (2D) ^13^C–^13^C dipolar-based MAS ssNMR experiments, where cross-peak correlations arise from pairs of NMR-active nuclei in spatial proximity. Depending on experimental parameters and conditions, ^13^C pairs with internuclear distances up to approximately 8 Å can potentially give rise to spectrally detectable cross-peaks.^40,45,46^ Dipolar-assisted rotational resonance (DARR^47^) and combined *R*2*_n_^v^*-driven (CORD^48^) experiments were conducted at various mixing times (15–300 ms, see SI Tables S1, S2, and S3). In addition, we conducted CHHC^49^ experiments that entail the advantage of direct transfer of polarization between protons that are close in space in folded regions of the polypeptide and have stronger dipolar couplings than ^13^C spin pairs.^50,51^ The 2D experiments were conducted on both fully and sparsely ^13^C-labeled samples of gVp in complex with unlabeled full-length ssDNA extracted directly from fd phage (fth1 strain, 8233 nucleotides^52^). Sparsely labeled samples, where only a subset of carbon sites are isotopically labeled, give rise to simplified, less crowded spectra with reduced effects of dipolar truncation and relayed polarization transfer, allowing better detection of long-range contacts.^53^ The resulting spectra also entail lower ambiguity levels on average, and therefore fewer assignments may be attributed to each cross-peak.

Cross-peak lists generated from eight spectra of the fully labeled sample, differing in experiment type, mixing time, and processing parameters, were concatenated into a single list. Similarly, 30 spectra of the sparsely labeled sample were combined. In order to generate a list of distance restraints, home-written Python scripts were provided with the two peak lists and the assigned chemical shifts of ssDNA-bound gVp as input. A chemical shift tolerance window of 0.3 ppm (a value typically chosen for ssNMR spectra^41,54^) was used in order to attribute all possible assignments to each cross-peak, resulting in a set of both ambiguous distance restraints (ADRs^55^) and unambiguous restraints (a single possible assignment). All restraints were set to a range of 2–8 Å.^40,56^

For restraints arising from the sparsely labeled data sets, we utilized a probability threshold of 40% in order to discard possible assignments, which corresponded to pairs of carbon sites that had a low probability of both being isotopically labeled. That is, we only considered an assignment to a cross-peak as plausible if the product of the probabilities of both carbon sites to be ^13^C-labeled in the sample was 40% or higher. Those statistics were derived using previously published statistical estimates for the effective probabilities of specific carbon sites in each type of amino acid to be ^13^C-labeled in a sample prepared with [1,3-^13^C]-glycerol as the sole carbon source.^53,57^

Both lists were further simplified by discarding restraints that had more than 20 possible assignments, as this was shown, in the context of solution NMR, to have the potential to improve the quality of the calculated model.^58,59^ An attempt to discard restraints that had more than five possible assignments resulted in failure to obtain a converged structure. An initial list of ADRs and non-ambiguous restraints was obtained by aggregating both restraint lists (fully and sparsely labeled) while giving precedence to restraints arising from the inherently less ambiguous, more informative sparse data, in spectral regions where the two peak-lists overlapped (see details regarding generation of distance restraints in the SI).

We applied several filters on the aggregated set of distance restraints in an attempt to discard restraints that do not arise from carbon–carbon correlations of structural significance, or those that are in significant violation of prior knowledge on the system. (1) We removed restraints that included a single-bond contact as one or more of the possible assignments, since such correlations are assumed to be much stronger than long-range contacts, and do not report on the overall fold of the protein. (2) We assumed that any pair of carbon sites further apart than 16 Å in the X-ray structure of free gVp will not be close enough in the ssDNA-bound form of the protein to result in a spectrally detectable cross-peak. While evidence previously reported by our lab has demonstrated that the structure of the gVp protein undergoes significant changes upon binding,^36^ we can safely assume that the complex assembly process does not entail a complete rearrangement of the overall fold.^33^ (3) We removed contacts with a high probability of association with the homodimer interface. NMR structure calculations of multimers are challenging due to the inability to a priori distinguish between cross-peaks arising from intra- and inter-monomer correlations.^60^ When attempting to calculate the monomer structure, any inter-monomer correlation arising from the dimer interface that will be erroneously designated as an intra-monomer contact would introduce conformational errors and therefore distort the elucidated fold of the monomer model. We therefore ruled out (using a home-written Python script) all restraints that included possible assignments corresponding to an inter-monomer distance shorter than 7 Å in the X-ray structure of the free gVp homodimer under the assumption that the dimer interfaces of the free and ssDNA-bound gVp dimer structures bear similarity. This assumption is further justified by findings previously reported by our lab, which demonstrated that the CSPs upon binding are relatively low in the dimer interface.^36^

The resulting set of restraints, after the application of the three filters, was the initial set provided as input to the calculation process. A total of 1901 distance restraints were provided as input to the initial step of the structural calculations, including 251 unambiguous restraints.

### Generation of Torsion Angle Restraints

The software tool TALOS+ was used in order to generate restraints on the ψ–ϕ torsion angles of the protein backbone.^37,61^ A total of 142 torsion angle restraints were deemed consistent by the software and were used as input for the subsequent calculation process.

### Structure Calculation

We used the Xplor-NIH^62^ software package iteratively to calculate the protein structure. Both the filtered distance restraints and the predicted angle restraints were provided as input, along with the protein sequence.

**Figure 2:**
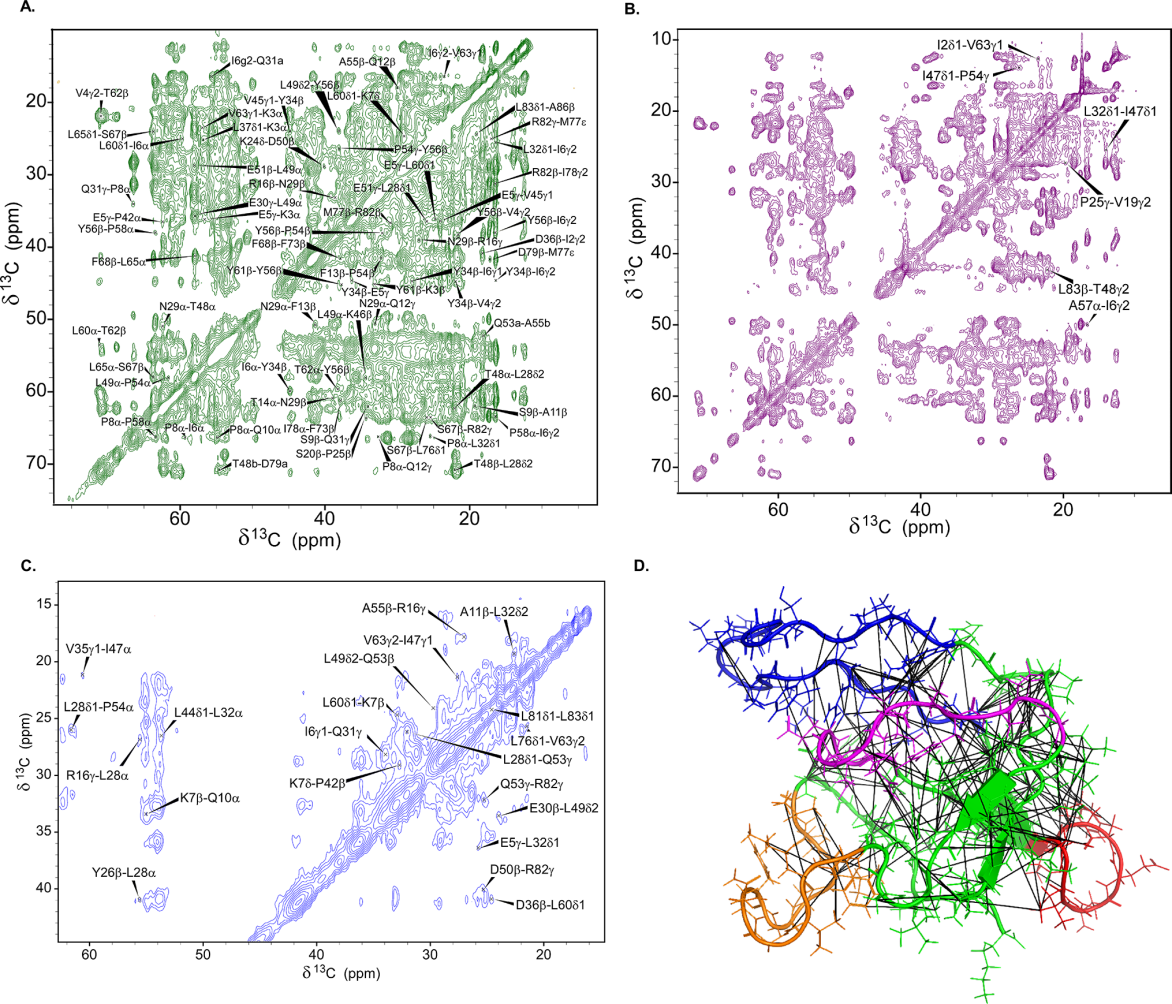
Solid-state NMR spectra depicting structurally informative unambiguous distance restraints. (A) DARR spectrum of sparsely (1,3-^13^C-glycerol) labeled gVp acquired with a mixing time of 300 ms. (B) DARR spectrum of fully labeled gVp acquired with a mixing time of 100 ms. (C) CHHC spectrum of sparsely labeled gVp acquired with a mixing time of 300 μs. All three spectra were apodized with a squared cosine bell function in both dimensions, and the lowest contour level was set to a signal-to-noise ratio of 7. (D) Unambiguous long-range distance restraints provided as input to the refinement step plotted as black lines on the ensemble-average structure. The four main loop regions are colored as in Figure 1.

During each iteration, a total of *N* structures (values used in the range of 100–250, see SI Table S5 for details) were generated. Afterward, these structures were sorted according to the energy score, and the *k* lowest energy structures (values used in the range of 10–25, Table S5) were further analyzed in order to update the restraints to be provided as input to the subsequent iteration. After each iteration, we used two filtration methods in order to better inform the set of restraints to be provided as input to the next Xplor-NIH run. First, distance and angle restraints that were significantly violated in a large subset of the top *k* lowest energy structures were removed (see Structure Calculation details in the SI). Second, the average structure of the *k* lowest energy structures was calculated by Xplor-NIH and any possible assignments corresponding to carbon pairs further apart than a specific cutoff distance (16 Å down to 8.5 Å) in the average structure were ruled out. In doing so, we treated the average structure of the previous run as a low-resolution model of the protein structure and accordingly modified the distance restraints for the next iteration. We conducted a total of 12 iterations of Xplor-NIH, with home-written Python scripts used intermittently for the restraint input modification described above (see Xplor scripts in the SI). Each iteration started from an arbitrary, extended conformation of the protein sequence, with ideal covalent geometry. Starting from an initial cutoff distance of 16 Å for distance restraint filtration from the 25-structure-average output of the first run, we gradually and iteratively decreased this filtration cutoff down to 8.5 Å (with *k* = 10), so that with each passing iteration, this filter on distance restraints was more strictly reliant on the outcome of the previous run. Revisions of the distance restraints list throughout the calculation process resulted in an iterative decrease to both their overall amount and to ambiguity levels (see Figure S3 in the SI). The final set of restraints included 112 torsion angle restraints and 1247 distance restraints, including 593 unambiguous restraints. Figure 2 displays several processed spectra, acquired with a variety of mixing times and pulse sequences, and illustrates cross-peaks that gave rise to unambiguous medium- and long-range restraints included in this final set.

Finally, the lowest energy structure of the 12th iteration was provided as an initial structure to a refinement process, conducted in implicit solvent.^63^

### Structure Validation

The average C*α*-RMSD of the final 10-structure ensemble with respect to their average structure is 1.2 Å across all 87 residues (see Figure S4). The backbone RMSD of the well-defined regions of the ensemble (residues 2–16, 28–37, and 41–87, as determined by the PDB structure validation report; this mainly excludes the DNA-binding loop) is 0.37 Å, well below the value of 2.0 Å, often used as the preliminary criterion for structural convergence (e.g., by the structure calculation software CS-Rosetta^64^).

In order to verify convergence, we used Xplor-NIH to conduct restraint violation analysis on the refined ensemble. Of the 1247 distance restraints and 112 angle restraints provided to the final refinement step, less than 2 and 7% of the distance and angle restraints, respectively, were violated (by more than 0.5 Å or 5^°^ for distance and angle restraints, respectively) in some or all members of the ensemble (see SI Tables S6–S9). Therefore, the refined ensemble is in good agreement with the final set of input restraints, with the vast majority of internuclear distances and torsion angles within the bounds of the experimentally derived and iteratively filtered constraints. Statistics based on the Ramachandran plot^65^ of the ψ–ϕ torsion angles of the ensemble indicated that none of the analyzed, well-defined residues (see Table S10 and Figure S5 in the SI) lie in the disallowed regions. This indicates that the majority of the protein backbone torsion angles are geometrically feasible in terms of sidechain steric hindrance. This finding is further corroborated by the MolProbity clash score,^66^ depicting the average number of steric atomic clashes larger than 0.4 Å per 1000 atoms (see details in the SI). The calculated ensemble clash score (calculated for well-defined residues, as determined by the PDB structure validation report) is 26, which is on par with MolProbity clash scores previously reported for other protein structures solved via MAS ssNMR (CAP-Gly bound to microtubules—PDB ID: 2MPX(41)–24; Crh dimer—PDB ID: 2RLZ(40)–27; GB1—PDB ID: 2KQ4(67)–28). The detailed structural statistics of the final ensemble are provided in Table 1.

**Table 1:** Structural Statistics

Distance restraints (input to refinement step)	
total	1247
average number of restraints per residue	14.3
average number of unambiguous long-range restraints per residue	3.2
unambiguous distance restraints	593
intra-residue (*i* = *j*)	80
sequential (|*i* – *j*| = 1)	112
medium range (1 < |*i* – *j*| < 5)	126
long-range (|*i* – *j*| ≥ 5)	275
total number of distance restraint violations (>0.5 Å)^a^	15
average number of distance restraint violations per structure (>0.5 Å)^a^	6.5
hydrogen-bond restraints	0
disulfide restraints	0
Dihedral angle restraints	
total	112
total number of dihedral angle violations^a^	7
average number of dihedral angle violations per structure^a^	2.4
ensemble validation parameters	
*Cα*-RMSD (all residues)^a^	1.2 Å
backbone RMSD (well-defined residues)^b^	0.37 Å
MolProbity clash score (well-defined residues)^b^	26
PROCHECK (ψ – ϕ; raw/*Z*-score)^c^	–0.82/–2.91
PROCHECK (all; raw/*Z*-score)^c^	–0.72/–4.26
Ramachandran statistics^c^	
most favored regions (%)	380 (77.6%)
additional allowed regions (%)	110 (22.4%)
generously allowed regions (%)	0 (0.0%)
disallowed regions (%)	0 (0.0%)

a Calculated with Xplor-NIH.

b *wwPDB validation report; calculated for well-defined regions (residues 2–16, 28–37, and 41–87).

c Protein Structure Validation Suite (calculated for ordered residues, defined as residue with dihedral angle order parameters obeying S(ϕ) + S(ψ) ≥1.8)— residues 3–13, 15–17, 27–30, 33–36, 41–49, 53–62, 68–77, and 81–86; Ramachandran statistics also exclude proline and glycine residues (out of a total of 87 residues, 57 are ordered, but five of which are proline resides and three are glycine residues; therefore, 49 residues are plotted for each ensemble member for a total of 490 data points).

### Characterization of the Calculated Ensemble

**Figure 3:**
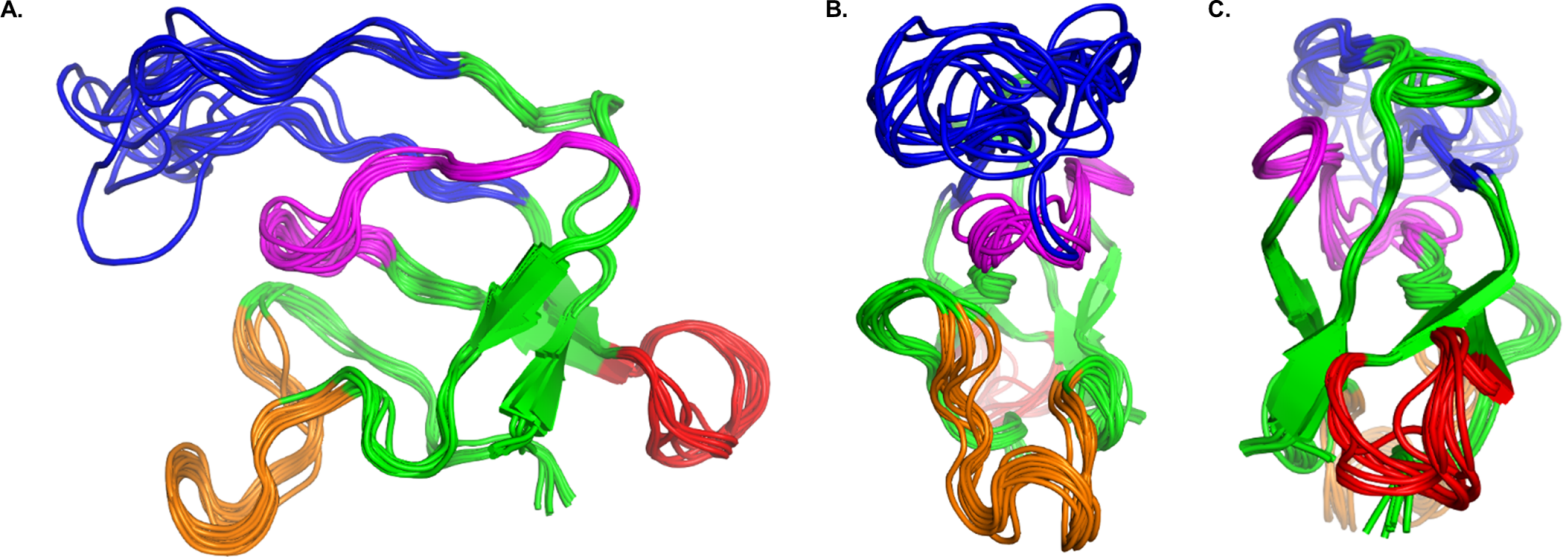
(A) The refined ensemble (10 structures, PDB ID 8ACZ) of ssDNA-bound gVp. The premature virion particle includes 8233 nucleotides and ∼2000 gVp protein subunits. The four main loops are colored as in Figure 1. (B) Rotation of the ensemble by 90^°^ around the vertical axis. (C) Rotation of the ensemble by 270^°^ around the vertical axis.

**Figure 4:**
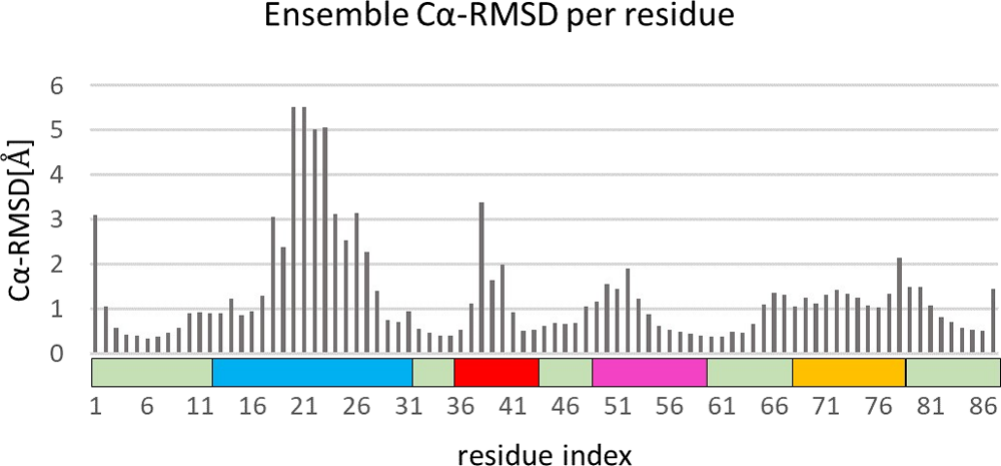
Cα-RMSD per residue, averaged across the 10 lowest energy structures of the final ensemble. Structural alignment and RMSD calculations were conducted via Chimera.^69^ The four main loop regions are colored as in Figure 1.

The 13-step structure calculation process resulted in a final refined ensemble of 10 structures shown in Figure 3, which we report as our model for the gVp monomer in complex with full-length, 8233-nucleotide-long ssDNA of fd phage. The structure was deposited to the Protein Data Bank with PDB ID 8ACZ. The Stride secondary structure determination algorithm^68^ detects four regions as beta strands in the ensemble-averaged structure—residues 2–4, 33–35, 43–46, and 61–63, located at the hydrophobic core. Both the complex loop (residues 36–43) and the dyad loop (residues 68–78) are relatively well-defined across the ensemble, with Cα-RMSD values of 1.3 Å (Figure 4). The core loop is also well-defined, with a Cα-RMSD of 1.0 Å.

Upon alignment of the ensemble structures, it can be seen that the orientation of the DNA-binding loop with respect to the hydrophobic core varies considerably, resulting in a high Cα-RMSD value of 2.5 Å. This variability may also be attributed to structural flexibility of the DNA binding loop with respect to the core, as previously reported.^23^ Such conformational plasticity may be beneficial for the binding of gVp dimers to ssDNA as part of the nucleoprotein complex assembly process. The high Cα-RMSD of this loop may also be explained by the long distance between the tip of the loop and the core regions of the protein, making it challenging to lock its orientation with respect to the rest of the protein using structural information composed of internuclear distances up to approximately 8 Å.

### Comparison of the Bound gVp Model to Free gVp

**Figure 5:**
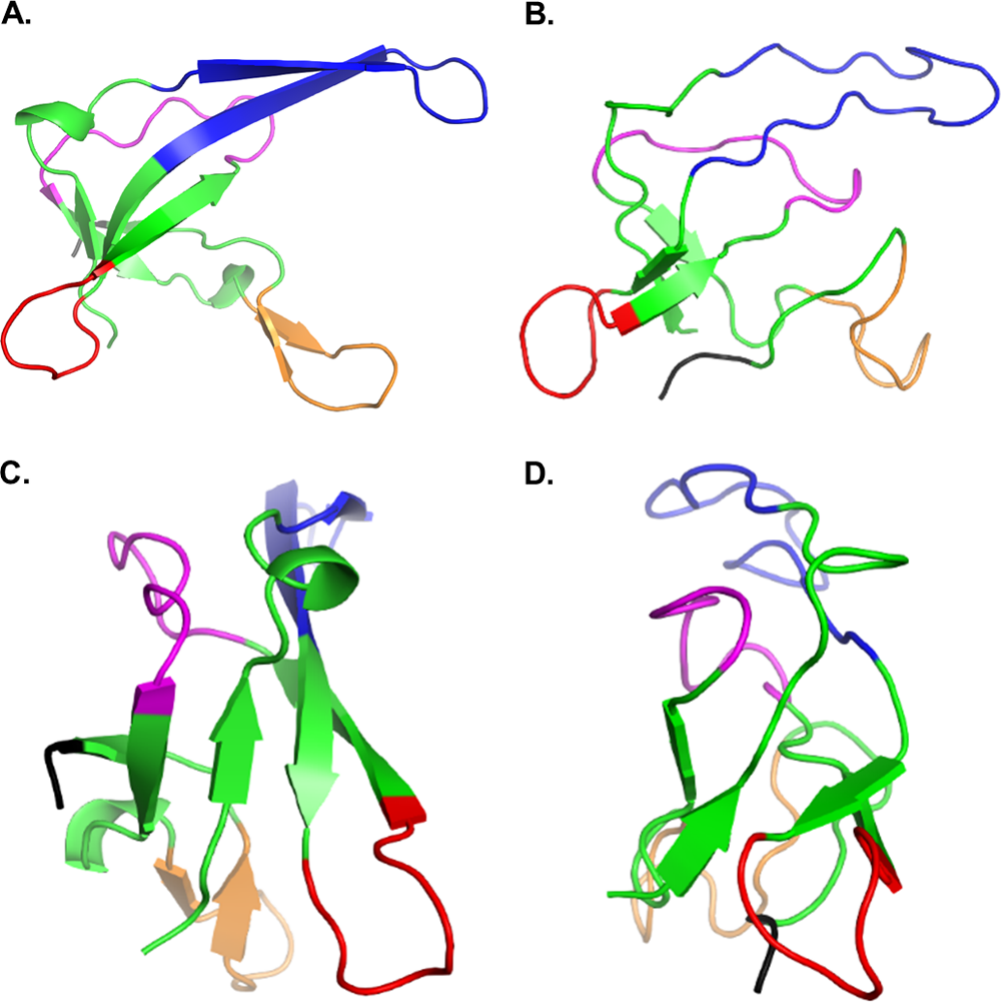
(A) X-ray structure of free gVp. (B) Ensemble-averaged ssNMR structure of ssDNA-bound gVp. (C) Rotation of the free gVp structure by 90^°^. (D) Rotation of the bound gVp structure by 90^°^. The C-terminus is colored in black, and the four main loops are colored as in Figure 1.

In a visual comparison of the ensemble-averaged structure of ssDNA-bound gVp with that of the X-ray structure of free gVp (Figure 5), it is apparent that the overall global fold is mostly conserved. The four main loops of free gVp (DNA, dyad, core, and complex) are all clearly visible in the bound form, even though some of their secondary structure elements, well-defined in the free form, are absent (described below). In addition, the beta sheet arrangement of the core of the monomer is mostly preserved. These results are in agreement with the assumption made during filtration of the initial set of distance restraints, that the tertiary structure is not changed altogether upon binding to phage ssDNA.

There are several significant structural changes detected throughout the sequence, reflected overall by a backbone (N, Co, C*α*) RMSD of 6.4 Å between the ensemble-averaged structure of bound gVp and the X-ray structure of free gVp. Such significant conformational changes accompanying the nucleoprotein complex assembly process are expected given the CSP analysis previously reported by our lab^36^ and agree well with the results reported here, as will be shown in detail.

Relative to the X-ray structure, the location of the C-terminus undergoes a significant structural change (Figure 5C,D). This final section of the sequence (residues 69–87) folds back toward an entirely different region of the protein. Some examples of unambiguous long-range contacts restricting this region (connecting carbon sites at the C-terminus region to carbon sites belonging to the hydrophobic core) are provided in the SI (Figure S7 and S8, Tables S11 and S12), corroborating this aspect of the calculated structure. Also, this result agrees well with the findings from the previous CSP results.^36^ The motional pathway for the transition between the free and bound gVp molecular conformations remains puzzling in terms of the change to the location of the C-terminus with respect to the core, and further research is required in order to decipher the trajectory of such a structural change.

**Figure 6:**
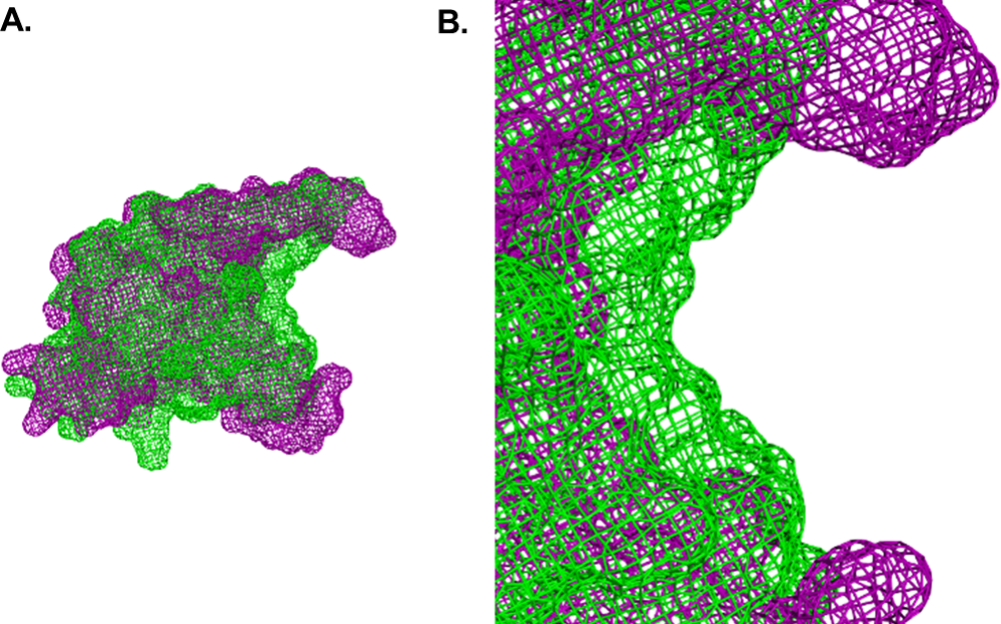
(A) Aligned overlay of the mesh representations of the free gVp monomer (X-ray structure, magenta) and the ensemble-averaged ssDNA-bound gVp ssNMR structure (green). (B) A closer view of the hypothesized DNA binding domain. The narrowing of the concave cleft upon binding is clearly visible.

Another major structural change relates to the concave clefts of each dimer structure, hypothesized to accommodate for the bound strands of the ssDNA. As mentioned, upon dimerization of two gVp monomers, two concave clefts are formed in the homodimer structure, each located between the dyad loop of one monomer and the DNA-binding loop of the other.^18^ As can be seen in our ensemble-averaged bound gVp structure, the available space between the DNA-binding loop and the dyad loop of the monomer is significantly decreased upon binding (Figures 5A,B and 6). This change may be described in terms of the proximity of both of these loops to the core loop. It is the decrease in both inter-loop distances (DNA-binding loop–core loop and dyad loop–core loop) that gives rise to a narrowing of the vacant volume between the loops that is available for the bound strands of nucleotides. Such a modification may facilitate the complexation of the nucleoprotein assembly. In order to quantify this change, we define an inter-loop distance as the average of all pairwise distances between all possible inter-loop pairs of C*α* carbons. Using this definition, averaged across all 10 members of the ensemble (see details in Table S13 in the SI), we calculated that the dyad loop–core loop inter-loop distance decreases by 6.6 Å (from 25.2 to 18.6 Å) upon binding, and the DNA-binding loop–core loop inter-loop distance decreases by 4.4 Å (from 18.4 to 14.0 Å). Once again, this finding is corroborated by the fact that both the DNA-binding loop and the core loop are expected to undergo significant structural changes, according to previously reported CSP analysis.^36^ While the results shown here indicate a narrowing of the cleft of a single monomer, if the twofold symmetry axis is preserved upon binding, then the observed approach of the DNA-binding loop and the dyad loop in an intra-monomer sense is expected to give rise to a shortening of two inter-monomer distances, each defined by the dyad loop of one monomer and the DNA-binding loop of the other monomer. This observation is significant, since it has long been reported that the binding of the dimer to the two anti-parallel strands of ssDNA involves the approach of two inter-monomer pairs of loops (DNA-binding loop and dyad loop) toward one another, with each such pair binding to one of the two strands forming the helical structure within the complex.^18^

**Figure 7:**
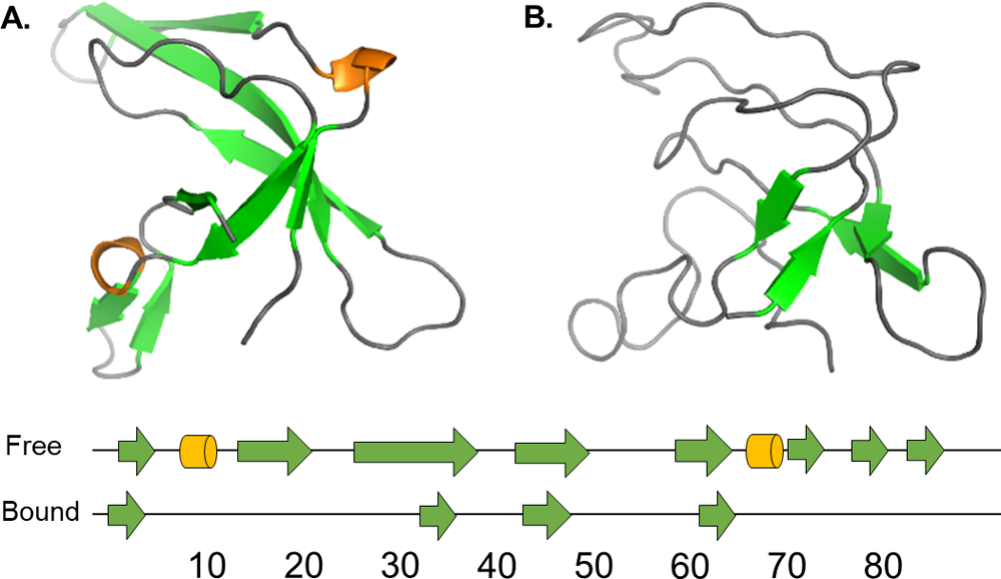
Secondary structure elements. (A) X-ray structure of free gVp. (B) Ensemble-averaged ssNMR structure of ssDNA-bound gVp. Beta strands are plotted in green, and 3_10_ helices are plotted in orange.

The free and bound gVp structures also differ in the recognition of secondary structure elements (Figure 7). According to the Stride^68^ algorithm, the two short 3_10_ helices reported in the free X-ray structure are no longer detected in the bound structure. Ramachandran analysis comparing the free and bound forms indicates that this discrepancy arises due to slight shifts of the corresponding residues in ψ–ϕ space (see Figure S6). Of the five beta strands comprising the beta barrel in the free form, four are partially detected in the bound model. These strands, recognized at the region most central to the hydrophobic core, form a short anti-parallel beta sheet, which is a subset of the beta barrel motif reported in the free structure. The fifth strand that is not detected at all in the bound form is located at the C-terminus of the free structure, where our model exhibits a significant change in tertiary structure (Figure 5C,D), and therefore, it is not surprising that this is accompanied by a change to the secondary structure. The DNA-binding loop protruding from the core includes the longest pair of anti-parallel beta-strands in the free gVp structure, and these strands, for the most part, are not detected in the bound form (only a short section of one of these strands, which is located at the core, at residues 43–46, is detected). It may be that the change in the orientation of the DNA-binding loop with respect to the core prevents the formation of hydrogen-bonded regions in both strands; therefore, the beta strands are no longer present in the bound form. While many residues corresponding to the beta-strand regions of free gVp do undergo small shifts in ψ–ϕ space, several residues do exhibit significant changes (see Figure S6). Since some of the beta-strands of free gVp are short, the residues that exhibit larger changes to their torsion angles, due to the tertiary changes throughout the structure, suffice to prevent determination of several regions as beta-strands.

The free form X-ray structure also includes a short anti-parallel beta ladder at the dyad loop (residues 68–70 and 76–78 in the free structure), which is not detected in the bound form by the Stride algorithm. In the bound structure, the conformation of both sections of the protein backbone with respect to one another is no longer viable for the formation of a set of hydrogen bonds necessary for formation of beta strands (Figure 5B). Yet, the location of the dyad loop (residues 68–78) with respect to the core is preserved overall.

**Figure 8:**
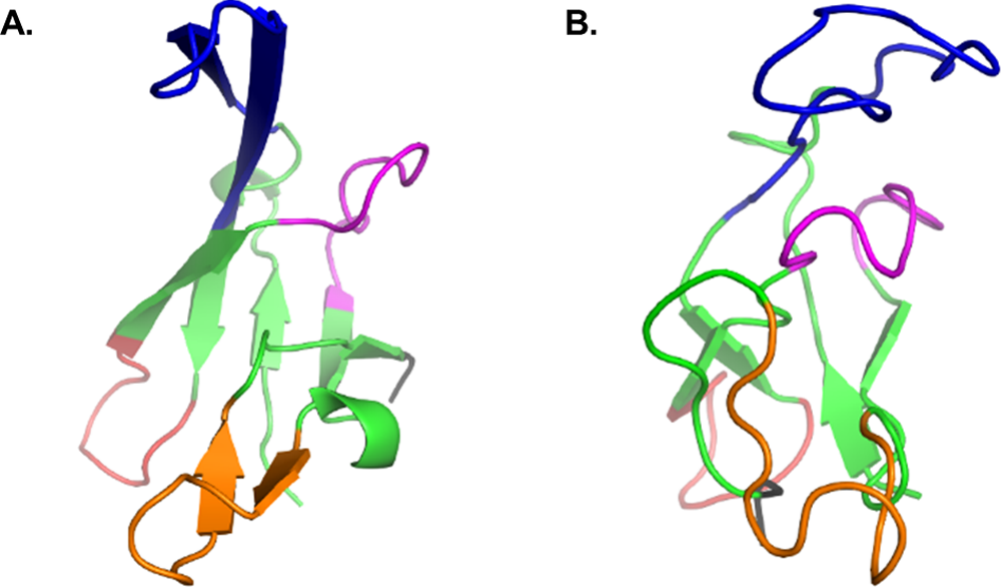
Rearrangement of the core loop. (A) X-ray structure of free gVp. (B) Ensemble-averaged ssNMR structure of ssDNA-bound gVp. Upon binding, the core loop (magenta) becomes aligned along the same vertical axis connecting the DNA-binding loop and the dyad loop. The color scheme is similar to Figure 1, and the view is as in Figure 3B.

Another interesting structural difference involves the alignment of the core loop with respect to the DNA-binding loop and the dyad loop (Figure 8). In the free gVp structure, this loop protrudes outward from the hydrophobic core, in an entirely different direction compared to the two loops, but in the bound model, the core loop is aligned along the same vertical axis as the DNA-binding loop and the dyad loop. This change in conformation may possibly be involved in the binding mechanism of the protein to the DNA. That is, this alignment of all three loops may be a result of their binding interactions with the ssDNA. It is also possible that such a re-ordering of the loops in 3D space is the result of a transition of the monomer to a more compact conformation, as the homodimers are arranged adjacently for the formation of the protein coat of the complex.

## Conclusions

We reported the first high-resolution 3D model for the structure of the gVp monomer in its conformation when bound to full-length ssDNA of the fd bacteriophage virus. Our model is directly based on restraints elucidated from experimental magic-angle spinning NMR data. The structure calculation protocol makes minimal use of the known free structure in order to improve the quality of the restraint input to the calculation of the bound form. It also takes measures in order to avoid conformational distortions that may arise from inter-monomer contacts of the homodimer.

This structure joins a relatively short list of non-crystalline protein structures that have been determined via MAS ssNMR in the context of their entire high-molecular-weight biological assembly, further demonstrating the power of this method in structural studies. As the use of AI methods for accurate determination of protein structure based solely on sequence is becoming prevalent, MAS ssNMR presents a tool to study specific structural changes involved in processes of DNA binding or complex assembly.

Results previously reported by our group have presented spectral evidence that gVp undergoes significant structural changes upon binding to ssDNA in the intracellular nucleoprotein complex. CSP analysis has detected regions of interest along the protein sequence, where such modifications to the conformation were expected to be most prominent. The structure reported here confirms the three main regions that were predicted to be most conformationally altered in the bound form: The DNA-binding loop protrudes from the core in a different angular conformation when compared to the free structure; the location of the C-terminus with respect to the hydrophobic core is changed, posing a large structural change with respect to the free protein: The core loop, which protrudes in a different direction from the DNA-binding loop and the dyad loop in the free form, is aligned with both loops in the bound form. These conformational changes seem to facilitate two processes: the binding to viral ssDNA, as the concave cleft where the ssDNA is hypothesized to be bound to the protein assumes a narrower conformation, and the cooperative binding interactions of dimers forming the capsid of the complex, as the entire protein assumes a more compact conformation.

## Supporting Information Available

Experimental parameters of NMR experiments; processing parameters of the spectral data; technical details on the generation of structural restraints; Xplor-NIH scripts; detailed results of the structural validation of the final ensemble. The Python scripts used for structure calculations available via a GitHub depository at https://github.com/YoavShamir5/Atomic-Resolution-Structure-of-the-Protein-Encoded-by-Gene-V-of-fd-Bacteriophage-in-Complex-. The raw NMR data are available via Zenodo, https://dx.doi.org/10.5281.zenodo.7294474 (PDF)

## References

[ref1] Marvin D. Filamentous Phage Structure, Infection and Assembly. Curr. Opin. Struct. Biol. 1998, 8, 150–158. 10.1016/S0959-440X(98)80032-8. 9631287

[ref2] Rakonjac J. Filamentous Bacteriophages: Biology and Applications. eLS 2012, 1.

[ref3] Hay I. D.; Lithgow T. Filamentous Phages: Masters of a Microbial Sharing Economy. EMBO Rep. 2019, 20, 1–24. 10.15252/embr.201847427. PMC6549030 30952693

[ref4] Rakonjac J.; Bennett N. J.; Spagnuolo J.; Gagic D.; Russel M. Filamentous Bacteriophage: Biology, Phage Display and Nanotechnology Applications. Curr. Issues Mol. Biol. 2011, 13, 51–76. 21502666

[ref5] Rasched I.; Oberer E. Ff Coliphages: Structural and Functional Relationships. Microbiol. Rev. 1986, 50, 401–427. 10.1128/mr.50.4.401-427.1986. 3540571 PMC373080

[ref6] Smeal S. W.; Schmitt M. A.; Pereira R. R.; Prasad A.; Fisk J. D. Simulation of the M13 Life Cycle II: Investigation of the Control Mechanisms of M13 Infection and Establishment of the Carrier State. Virology 2017, 500, 275–284. 10.1016/j.virol.2016.08.015. 27569186

[ref7] Zinder N. D.; Boeke J. D. The Filamentous Phage (Ff) as Vectors for Recombinant DNA- a Review. Gene 1982, 19, 1–10. 10.1016/0378-1119(82)90183-4. 6292041

[ref8] Hoffmann-Berling H.; Mazé R. Release of male-specific bacteriophages from surviving host bacteria. Virology 1964, 22, 305–313. 10.1016/0042-6822(64)90021-2. 14127828

[ref9] Loeb T. Isolation of a Bacteriophage Specific for the F+ and Hfr Mating Types of Escherichia coli K-12. Science 1960, 131, 932–933. 10.1126/science.131.3404.932. 14417842

[ref10] Marvin D. A.; Hoffman-Berling H. Physical and Chemical Properties of Two New Small Bacteriophages. Nature 1963, 197, 517–518. 10.1038/197517b0.

[ref11] Székely A. J.; Breitbart M. Single-Stranded DNA Phages: From Early Molecular Biology Tools to Recent Revolutions in Environmental Microbiology. FEMS Microbiol. Lett. 2016, 363, 1–9. 10.1093/femsle/fnw027. 26850442

[ref12] Goldbourt A. Magic-Angle Spinning NMR of Bacteriophage Viruses. eMagRes 2015, 9, 173–182. 10.1016/j.pnmrs.2015.02.003.

[ref13] Day L. A.; Inoviruses, in Encyclopedia of Virology; Edition 3; Mahy B. W. J.; Van Regenmortel M. H. V., Eds.; Elsevier, Oxford, 2008, pp. 117–124.

[ref14] Petersen G. B.; Hill D. F. Nucleotide Sequence of Bacteriophage F1 DNA. J. Virol. 1982, 44, 32–46. 10.1128/jvi.44.1.32-46.1982. 6292494 PMC256238

[ref15] Clarke M.; Maddera L.; Harris R. L.; Silverman P. M. F-Pili Dynamics by Live-Cell Imaging. Proc. Natl. Acad. Sci. U. S. A. 2008, 105, 17978–17981. 10.1073/pnas.0806786105. 19004777 PMC2582581

[ref16] Costa T. R. D.; Ilangovan A.; Ukleja M.; Redzej A.; Santini J. M.; Smith T. K.; Egelman E. H.; Waksman G. Structure of the Bacterial Sex F Pilus Reveals an Assembly of a Stoichiometric Protein-Phospholipid Complex. Cell 2016, 166, 1436–1444.e10. 10.1016/j.cell.2016.08.025. 27610568 PMC5018250

[ref17] Lubkowski J.; Hennecke F.; Plückthun A.; Wlodawer A. Filamentous Phage Infection: Crystal Structure of G3p in Complex with Its Coreceptor, the C-Terminal Domain of TolA. Structure 1999, 7, 711–722. 10.1016/S0969-2126(99)80092-6. 10404600

[ref18] Stassen A. P.; Folmer R. H.; Hilbers C. W.; Konings R. N. Single-Stranded DNA Binding Protein Encoded by the Filamentous Bacteriophage M13: Structural and Functional Characteristics. Mol. Biol. Rep. 1994, 20, 109–127. 10.1007/BF00990543. 7565651

[ref19] Gray C. W. Three-Dimensional Structure of Complexes of Single-Stranded DNA-Binding Proteins with DNA. IKe and Fd Gene 5 Proteins Form Left-Handed Helices with Single-Stranded DNA. J. Mol. Biol. 1989, 208, 57–64. 10.1016/0022-2836(89)90087-9. 2671388

[ref20] Alma N. C. M.; Harmsen B. J. M.; de Jong E. A. M.; Ven J. v. d.; Hilbers C. W. Fluorescence Studies of the Complex Formation between the Gene 5 Protein of Bacteriophage M13 and Polynucleotides. J. Mol. Biol. 1983, 163, 47–62. 10.1016/0022-2836(83)90029-3. 6601193

[ref21] Oey J. L.; Knippers R. Properties of the Isolated Gene 5 Protein of Bacteriophage Fd. J. Mol. Biol. 1972, 68, 125–138. 10.1016/0022-2836(72)90268-9. 4559110

[ref22] Skinner M. M.; Zhangt H.; Leschnitzer D. H.; Guant Y.; Bellamyt H.; Sweet R. M.; Gray C. W.; Koningsii R. N.; Wangt A. H.; Terwilliger T. C. Structure of the Gene V Protein of Bacteriophage Fl Determined by Multiwavelength X-Ray Diffraction on the Selenomethionyl Protein. Proc. Natl. Acad. Sci. U. S. A. 1994, 91, 2071–2075. 10.1073/pnas.91.6.2071. 8134350 PMC43311

[ref23] Folkers P. J. M.; Nilges M.; Folmer R. H. A.; Konings R. N. H.; Hilbers C. W. The Solution Structure of the Tyr41 → His Mutant of the Single-Stranded DNA Binding Protein Encoded by Gene V of the Filamentous Bacteriophage M13. J. Mol. Biol. 1994, 229–246. 10.1006/jmbi.1994.1132. 8107108

[ref24] Prompers J. J.; Folmer R. H. A.; Nilges M.; Folkers P. J. M.; Konings R. N. H.; Hilbers C. W. Refined Solution Structure of the Tyr41→His Mutant of the M13 Gene V Protein: A Comparison with the Crystal Structure. Eur. J. Biochem. 1995, 232, 506–514. 10.1111/j.1432-1033.1995.506zz.x. 7556200

[ref25] Guan Y.; Zhang H.; Wang A. H.-J.; Konings R. N. H.; Hilbers C. W.; Terwilliger T. C. Crystal Structures of Y41H and Y41F Mutants of Gene V Protein from Ff Phage Suggest Possible Protein-Protein Interactions in the GVP-ssDNA Complex. Biochemistry 1994, 33, 7768–7778. 10.1021/bi00191a004. 8011642

[ref26] Folkers P. J. M.; van Duynhoven J. P. M.; Jonker A. J.; Harmsen B. J. M.; Konings R. N. H.; Hilbers C. W. Sequence-specific ^1^H-NMR Assignment and Secondary Structure of the Tyr41 → His Mutant of the Single-stranded DNA Binding Protein, Gene V Protein, Encoded by the Filamentous Bacteriophage M13. Eur. J. Biochem. 1991, 202, 349–360. 10.1111/j.1432-1033.1991.tb16382.x. 1761038

[ref27] Murzin A. G. OB(Oligonucleotide/Oligosaccharide Binding)-Fold: Common Structural and Functional Solution for Non-Homologous Sequences. EMBO J. 1993, 12, 861–867. 10.1002/j.1460-2075.1993.tb05726.x. 8458342 PMC413284

[ref28] Konings R. N. H.; Folmer R. H. A.; Folkers P. J. M.; Nilges M.; Hilbers C. W. Three-Dimensional Structure of the Single-Stranded DNA-Binding Protein Encoded by Gene V of the Filamentous Bacteriophage M13 and a Model of Its Complex with Single-Stranded DNA. FEMS Microbiol. Rev. 1995, 17, 57–72. 10.1111/j.1574-6976.1995.tb00188.x.

[ref29] Folkers P. J. M.; van Duynhoven J. P. M.; van Lieshout H. T. M.; Harmsen B. J. M.; Konings R. N. H.; Hilbers C. W.; van Boom J. H.; Tesser G. I. Exploring the DNA Binding Domain of Gene V Protein Encoded by Bacteriophage M13 with the Aid of Spin-Labeled Oligonucleotides in Combination with ^1^H-NMR. Biochemistry 1993, 32, 9407–9416. 10.1021/bi00087a020. 8396429

[ref30] Van Duynhoven J. P. M.; Nooren I. M. A.; Swinkels D. W.; Folkers P. J. M.; Harmsen B. J. M.; Konings R. N. H.; Tesser G. I.; Hilbers C. W. Exploration of the Single-stranded DNA-binding Domains of the Gene V Proteins Encoded by the Filamentous Bacteriophages IKe and M13 by Means of Spin-labeled Oligonucleotide and Lanthanide-chelate Complexes. Eur. J. Biochem. 1993, 216, 507–517. 10.1111/j.1432-1033.1993.tb18169.x. 8375389

[ref31] Guan Y.; Zhang H.; Wang A. H. Electrostatic Potential Distribution of the Gene V Protein from Ff Phage Facilitates Cooperative DNA Binding: A Model of the GVP-ssDNA Complex. Protein Sci. 1995, 4, 187–197. 10.1002/pro.5560040206. 7757008 PMC2143068

[ref32] King G. C.; Coleman J. E. Two-Dimensional 1H NMR of Gene 5 Protein Indicates That Only Two Aromatic Rings Interact Significantly with Oligodeoxynucleotide Bases. Biochemistry 1987, 26, 2929–2937. 10.1021/bi00384a039. 3606999

[ref33] Folmer R. H.; Nilges M.; Folkers P. J.; Konings R. N.; Hilbers C. W. A Model of the Complex between Single-Stranded DNA and the Single-Stranded DNA Binding Protein Encoded by Gene V of Filamentous Bacteriophage M13. J. Mol. Biol. 1989, 240, 341–357. 10.1006/jmbi.1994.1449. 8035458

[ref34] Lecoq L.; Fogeron M. L.; Meier B. H.; Nassal M.; Böckmann A. Solid-State NMR for Studying the Structure and Dynamics of Viral Assemblies. Viruses 2020, 12, 1–26. 10.3390/v12101069. PMC7599928 32987909

[ref35] Hassid R. R.; Kedem S.; Bachar-Beck M.; Shamir Y.; Goldbourt A. Solid State NMR Chemical Shift Assignment of the Non-Structural Single-Stranded DNA Binding Protein gVp from fd Bacteriophage. Biomol. NMR Assignments 2022, 1–5. 10.1007/s12104-022-10076-5. 35460051

[ref36] Kedem S.; Hassid R. R.; Shamir Y.; Goldbourt A. Conformational Changes in Ff Phage Protein GVp upon Complexation with Its Viral Single-Stranded DNA Revealed Using Magic-Angle Spinning Solid-State NMR. Viruses 2022, 14, 1264 10.3390/v14061264. 35746735 PMC9231167

[ref37] Cornilescu G.; Delaglio F.; Bax A. Protein Backbone Angle Restraints from Searching a Database for Chemical Shift and Sequence Homology. J. Biomol. NMR 1999, 13, 289–302. 10.1023/A:1008392405740. 10212987

[ref38] Franks W. T.; Wylie B. J.; Schmidt H. L. F.; Nieuwkoop A. J.; Mayrhofer R. M.; Shah G. J.; Graesser D. T.; Rienstra C. M. Dipole Tensor-Based Atomic-Resolution Structure Determination of a Nanocrystalline Protein by Solid-State NMR. Proc. Natl. Acad. Sci. U. S. A. 2008, 105, 4621–4626. 10.1073/pnas.0712393105. 18344321 PMC2290771

[ref39] Sengupta I.; Nadaud P. S.; Jaroniec C. P. Protein Structure Determination with Paramagnetic Solid-State NMR Spectroscopy. Acc. Chem. Res. 2013, 46, 2117–2126. 10.1021/ar300360q. 23464364

[ref40] Loquet A.; Bardiaux B.; Gardiennet C.; Blanchet C.; Baldus M.; Nilges M.; Malliavin T.; Böckmann A. 3D Structure Determination of the Crh Protein from Highly Ambiguous Solid-State NMR Restraints. J. Am. Chem. Soc. 2008, 130, 3579–3589. 10.1021/ja078014t. 18284240

[ref41] Yan S.; Guo C.; Hou G.; Zhang H.; Lu X.; Williams J. C.; Polenova T. Atomic-Resolution Structure of the CAP-Gly Domain of Dynactin on Polymeric Microtubules Determined by Magic Angle Spinning NMR Spectroscopy. Proc. Natl. Acad. Sci. U. S. A. 2015, 112, 14611–14616. 10.1073/pnas.1509852112. 26604305 PMC4664305

[ref42] Tuttle M. D.; Comellas G.; Nieuwkoop A. J.; Covell D. J.; Berthold D. A.; Kloepper K. D.; Courtney J. M.; Kim J. K.; Barclay A. M.; Kendall A.; Wan W.; Stubbs G.; Schwieters C. D.; Lee V. M. Y.; George J. M.; Rienstra C. M. Solid-State NMR Structure of a Pathogenic Fibril of Full-Length Human α-Synuclein. Nat. Struct. Mol. Biol. 2016, 23, 409–415. 10.1038/nsmb.3194. 27018801 PMC5034296

[ref43] Daskalov A.; El Mammeri N.; Lends A.; Shenoy J.; Lamon G.; Fichou Y.; Saad A.; Martinez D.; Morvan E.; Berbon M.; Grélard A.; Kauffmann B.; Ferber M.; Bardiaux B.; Habenstein B.; Saupe S. J.; Loquet A. Structures of Pathological and Functional Amyloids and Prions, a Solid-State NMR Perspective. Front. Mol. Neurosci. 2021, 14, 1–18. 10.3389/fnmol.2021.670513. PMC8280340 34276304

[ref44] Wang S.; Munro R. A.; Shi L.; Kawamura I.; Okitsu T.; Wada A.; Kim S. Y.; Jung K. H.; Brown L. S.; Ladizhansky V. Solid-State NMR Spectroscopy Structure Determination of a Lipid-Embedded Heptahelical Membrane Protein. Nat. Methods 2013, 10, 1007–1012. 10.1038/nmeth.2635. 24013819

[ref45] Goldbourt A. Biomolecular Magic-Angle Spinning Solid-State NMR: Recent Methods and Applications. Curr. Opin. Biotechnol. 2013, 24, 705–715. 10.1016/j.copbio.2013.02.010. 23481376

[ref46] Shcherbakov A. A.; Medeiros-Silva J.; Tran N.; Gelenter M. D.; Hong M. From Angstroms to Nanometers: Measuring Interatomic Distances by Solid-State NMR. Chem. Rev. 2022, 122, 9848–9879. 10.1021/acs.chemrev.1c00662. 34694769 PMC9035484

[ref47] Takegoshi K.; Nakamura T.; Terao T. ^13^C-^1^H Dipolar-Assisted Rotational Resonance in Magic-Angle Spinning NMR. Chem. Phys. Lett. 2001, 344, 631–637. 10.1016/S0009-2614(01)00791-6.

[ref48] Hou G.; Yan S.; Trébosc J.; Amoureux J. P.; Polenova T. Broadband Homonuclear Correlation Spectroscopy Driven by Combined *R*2*_n_^v^* Sequences under Fast Magic Angle Spinning for NMR Structural Analysis of Organic and Biological Solids. J. Magn. Reson. 2013, 232, 18–30. 10.1016/j.jmr.2013.04.009. 23685715 PMC3703537

[ref49] Wilhelm M.; Feng H.; Tracht U.; Spiess H. W. 2D CP/MAS 13C Isotropic Chemical Shift Correlation Established by 1H Spin Diffusion. J. Magn. Reson. 1998, 134, 255–260. 10.1006/jmre.1998.1512. 9761701

[ref50] Korukottu J.; Schneider R.; Vijayan V.; Lange A.; Pongs O.; Becker S.; Baldus M.; Zweckstetter M. High-Resolution 3D Structure Determination of Kaliotoxin by Solid-State NMR Spectroscopy. PLoS One 2008, 3, e2359 10.1371/journal.pone.0002359. 18523586 PMC2387072

[ref51] Aluas M.; Tripon C.; Griffin J. M.; Filip X.; Ladizhansky V.; Griffin R. G.; Brown S. P.; Filip C. CHHC and 1H-1H Magnetization Exchange: Analysis by Experimental Solid-State NMR and 11-Spin Density-Matrix Simulations. J. Magn. Reson. 2009, 199, 173–187. 10.1016/j.jmr.2009.04.013. 19467890 PMC2706310

[ref52] Enshell-Seijffers D.; Smelyanski L.; Gershoni J. M. The Rational Design of a “type 88” Genetically Stable Peptide Display Vector in the Filamentous Bacteriophage Fd. Nucleic Acids Res. 2001, 29, 50e 10.1093/nar/29.10.e50. PMC55471 11353095

[ref53] Castellani F.; van Rossum B.; Diehl A.; Schubert M.; Rehbein K.; Oschkinat H. Structure of a Protein Determined by Solid-State Magic-Angle-Spinning NMR Spectroscopy. Nature 2002, 420, 99–102. 10.1038/nature01070. 12422222

[ref54] Lu M.; Russell R. W.; Bryer A. J.; Quinn C. M.; Hou G.; Zhang H.; Schwieters C. D.; Perilla J. R.; Gronenborn A. M.; Polenova T. Atomic-Resolution Structure of HIV-1 Capsid Tubes by Magic-Angle Spinning NMR. Nat. Struct. Mol. Biol. 2020, 27, 863–869. 10.1038/s41594-020-0489-2. 32901160 PMC7490828

[ref55] Nilges M. Calculation of Protein Structures with Ambiguous Distance Restraints. Automated Assignment of Ambiguous NOE Crosspeaks and Disulphide Connectivities. J. Mol. Biol. 1995, 245, 645–660. 10.1006/jmbi.1994.0053. 7844833

[ref56] Morag O.; Sgourakis N. G.; Abramov G.; Goldbourt A. Filamentous Bacteriophage Viruses: Preparation, Magic-Angle Spinning Solid-State NMR Experiments, and Structure Determination in Protein NMR, In Methods in Molecular Biology, Vol. 1688; Ghose R.; Humana Press, New York, NY, 2017, pp. 67–97. 10.1007/978-1-4939-7386-6_4 29151205

[ref57] Higman V. A.; Flinders J.; Hiller M.; Jehle S.; Markovic S.; Fiedler S.; van Rossum B. J.; Oschkinat H. Assigning Large Proteins in the Solid State: A MAS NMR Resonance Assignment Strategy Using Selectively and Extensively 13C-Labelled Proteins. J. Biomol. NMR 2009, 44, 245–260. 10.1007/s10858-009-9338-7. 19609683

[ref58] Habeck M.; Rieping W.; Linge J. P.; Nilges M. NOE Assignment with ARIA 2.0: The Nuts and Bolts. Methods Mol. Biol. 2004, 278, 379–402. 10.1385/1-59259-809-9:379. 15318004

[ref59] Linge J. P.; Habeck M.; Rieping W.; Nilges M. ARIA: Automated NOE Assignment and NMR Structure Calculation. Bioinformatics 2003, 19, 315–316. 10.1093/bioinformatics/19.2.315. 12538267

[ref60] O’Donoghue S. I.; King G. F.; Nilges M. Calculation of Symmetric Multimer Structures from NMR Data Using a Priori Knowledge of the Monomer Structure, Co-Monomer Restraints, and Interface Mapping: The Case of Leucine Zippers. J. Biomol. NMR 1996, 8, 193–206. 10.1007/BF00211165. 22911141

[ref61] Shen Y.; Delaglio F.; Cornilescu G.; Bax A. TALOS+: A Hybrid Method for Predicting Protein Backbone Torsion Angles from NMR Chemical Shifts. J. Biomol. NMR 2009, 44, 213–223. 10.1007/s10858-009-9333-z. 19548092 PMC2726990

[ref62] Bermejo G. A.; Schwieters C. D.; Protein Structure Elucidation from NMR Data with the Program Xplor-NIH in Protein NMR, In Methods in Molecular Biology, Vol. 1688; Ghose R.; Humana Press, New York, NY, 2017, pp. 67–97. 10.1007/978-1-4939-7386-6_14 PMC6771931 29151215

[ref63] Chen J.; Im W.; Brooks C. L. Refinement of NMR Structures Using Implicit Solvent and Advanced Sampling Techniques. J. Am. Chem. Soc. 2004, 126, 16038–16047. 10.1021/ja047624f. 15584737

[ref64] Shen Y.; Lange O.; Delaglio F.; Rossi P.; Aramini J. M.; Liu G.; Eletsky A.; Wu Y.; Singarapu K. K.; Lemak A.; Ignatchenko A.; Arrowsmith C. H.; Szyperski T.; Montelione G. T.; Baker D.; Bax A. Consistent Blind Protein Structure Generation from NMR Chemical Shift Data. Proc. Natl. Acad. Sci. U. S. A. 2008, 105, 4685–4690. 10.1073/pnas.0800256105. 18326625 PMC2290745

[ref65] Ramakrishnan C.; Ramachandran G. N. Stereochemical Criteria for Polypeptide and Protein Chain Conformations: II. Allowed Conformations for a Pair of Peptide Units. Biophys. J. 1965, 5, 909–933. 10.1016/S0006-3495(65)86759-5. 5884016 PMC1367910

[ref66] Williams C. J.; Headd J. J.; Moriarty N. W.; Prisant M. G.; Videau L. L.; Deis L. N.; Verma V.; Keedy D. A.; Hintze B. J.; Chen V. B.; Jain S.; Lewis S. M.; Arendall W. B. III; Snoeyink J.; Adams P. D.; Lovell S. C.; Richardson J. S.; Richardson D. C. MolProbity: More and Better Reference Data for Improved All-Atom Structure Validation. Protein Sci. 2018, 27, 293–315. 10.1002/pro.3330. 29067766 PMC5734394

[ref67] Nieuwkoop A. J.; Wylie B. J.; Franks W. T.; Shah G. J.; Rienstra C. M. Atomic Resolution Protein Structure Determination by Three-Dimensional Transferred Echo Double Resonance Solid-State Nuclear Magnetic Resonance Spectroscopy. J. Chem. Phys 2009, 131, 095101 10.1063/1.3211103. 19739873 PMC2832044

[ref68] Heinig M.; Frishman D. STRIDE: A Web Server for Secondary Structure Assignment from Known Atomic Coordinates of Proteins. Nucleic Acids Res. 2004, 32, w500–w502. 10.1093/nar/gkh429. 15215436 PMC441567

[ref69] Pettersen E. F.; Goddard T. D.; Huang C. C.; Couch G. S.; Greenblatt D. M.; Meng E. C.; Ferrin T. E. UCSF Chimera - A Visualization System for Exploratory Research and Analysis. J. Comput. Chem. 2004, 25, 1605–1612. 10.1002/jcc.20084. 15264254

