# Supplementary material for: Atomic-Resolution Structure of the Protein Encoded by Gene V of fd Bacteriophage in Complex with Viral ssDNA Determined by Magic-Angle Spinning Solid-State NMR
Source: J Am Chem Soc. 2022 Dec 21;145(1):300–10. doi: 10.1021/jacs.2c09957 (PMC9837838; doi:10.1021/jacs.2c09957)
Supplement: Supplementary file 1 — ja2c09957_si_001.pdf [file ja2c09957_si_001.pdf]

Supporting Information for

Atomic-Resolution Structure of the Protein Encoded by Gene V of fd Bacteriophage in Complex with Viral ssDNA Determined by Magic-Angle Spinning Solid-State NMR

Yoav Shamir and Amir Goldbourt*

School of Chemistry, Tel Aviv University, Tel Aviv 6997801, Israel

**amirgo@tauex.tau.ac.il*

Table of contents

Amino acid sequence of gVp 3
Sample preparation 3
NMR spectroscopy 3
Data processing 3
Experimental parameters 4
Processing parameters 7
Generation of peak lists 9
 Contour level adjustment 9
Automated peak-picking 9
 Filtration of spinning-sidebands and diagonal peaks 10
Generation of distance restraints 11
 Initial elucidation of restraints based on peak lists 11
 Aggregation of distance restraints across fully and sparsely labeled samples 11
 Distance restraint filtration 11
Structure calculation protocol 12
Iteration details 13
Xplor-NIH scripts 14
 Structure generation script 14
 Refinement script 16
Restraints input to refinement step 18
Violation analysis in the refined ensemble 18
Distribution of distance restraints by ambiguity level throughout the calculation process 20
Cα-RMSD and energy score throughout the calculation process 21
Ensemble validation 22
 Ramachandran plot of torsion angles 22
Ramachandran plot comparison of free and bound gVp 23
MolProbity clashscore 23
Unambiguous long-range restraints at regions of interest 24
 Location of C-terminus with respect to the core 24
 Proximity of dyad loop and core loop 25
Inter-loop distances 26
References 27

Amino acid sequence of gVp

The protein sequences of the three Ff phages (fd, f1, M13) are similar and consist of 87 residues. The sample used in this research included an additional 12-residue-long his-tag sequence at the C-terminus (sequence: LAAALEHHHHHH). This tag is NMR-invisible.

MIKVEIKPSQAQFTTRSGVSRQGKPYSLNEQLCYVDLGNEYPVLVKITLDEGQPAYAPGLYTVHLSSFKVGQFGSLMIDRLRLVPAK

Sample preparation

Samples of both U-[^{13}C,^{15}N]-glucose (fully labeled) and [1,3-^{13}C]-glycerol (sparsely labeled) gVp in complex with unlabeled ssDNA of fd phage were prepared as reported elsewhere[1] by mixing gVp proteins, recombinantly expressed with one of two isotopic labeling schemes and purified[2], with viral ssDNA extracted from intact fd phage samples. We used a ratio of four nucleotides per gVp monomer in accordance with literature[3]. Samples were packed into 4mm ZrO_2 MAS NMR rotors.

NMR spectroscopy

NMR experiments were conducted on a Bruker Avance III spectrometer operating at a magnetic field of 14.1T using a wide-bore 4mm Efree probe or a standard 4mm probe with a solenoid coil, both operating in double resonance ^1H-^{13}C mode. Sample temperature was maintained at $-10° C$ at the entrance to the stator. Two-dimensional ^{13}C-^{13}C correlation experiments were acquired using DARR[4], CORD[5], and CHHC[6] pulse sequences conducted with a variety of mixing times. Explicit experimental parameters are provided in detail in Table S1, S2 and S3.

All experimental data sets were processed via NmrPipe[7] using the NMRbox platform[8]. In order to maximize the number of cross-peaks that may be extracted from the entire spectral range, within the empirical limits of sensitivity and resolution, we applied several different combinations of apodization functions on both frequency dimensions (Table S4). Overall, we used 8 processed spectra of the uniformly labeled sample, and 30 of the sparsely labeled sample, each is the outcome of a combination of the pulse sequence used and the processing method employed.

Data processing

The spectral data were processed via NMRPipe[7]. Manual phase correction was conducted along with polynomial baseline adjustment. For each of the DARR and CORD experiments we used the following four different processing methods utilizing different combinations of apodization functions in order to maximize the number of peaks that could be extracted from each 2D time-domain data set (see Tables S4 for details).

- 200[Hz] exponential function for both dimensions.
- Squared cosine bell function for both dimensions.
- Squared cosine bell for the direct dimension and 200[Hz] exponential function for the indirect dimension.
- 100[Hz] exponential function for the direct dimension and 200[Hz] exponential function for the indirect dimension.

A single processing method was used for the CHHC experiments (Table S4).

Experimental parameters

Table S1. Experimental parameters of DARR experiments.

Experiment number	1	2	3	4	5	6	7
Probe	4mm Efree	4mm Efree	4mm Efree	4mm Efree	4mm Efree	4mm solenoid	4mm Efree
Carbon isotopic labelling	$^{13}C_6$-glucose	$^{13}C_6$- glucose	[1,3-^{13}C]-glycerol	[1,3-^{13}C]-glycerol	[1,3-^{13}C]-glycerol	[1,3-^{13}C]-glycerol	[1,3-^{13}C]-glycerol
^{1}H Frequency [MHz]	599.8	599.8	599.8	599.8	599.8	599.8	599.8
MAS rate (ω_r) [kHz]	13.000	13.000	13.000	13.000	13.000	13.000	13.000
Set temperature [°C]	-10.0	-10.0	-10.0	-10.0	-10.0	-10.0	-10.0
Carrier frequency [ppm]	100.2	100.2	100.0	100.0	100.0	101.9	103.4
Mixing time [ms]	15	100	50	100	300	150	300
Acquisition points (t_1/t_2)	1024/5988	1024/5988	1024/5988	1024/5988	1400/5988	600/4990	620/4990
Acquisition times (t_1/t_2) [ms]	13.11/29.94	13.13/29.94	13.13/29.94	13.13/29.94	17.95/29.94	7.79/24.95	8.05/24.95
Acquisition mode	States	States	States	States	States	States	States
$H90[\mu s]$	2.75	2.75	2.63	2.63	2.63	3.75	2.9
$C90[\mu s]$	5.00	5.00	5.00	5.00	5.00	4.5	5.2
CP rf power (v_H/v_C) [kHz]	79/50	79/50	68/50	68/50	68/50	47/50	63/48
CP contact time [ms]	1.8	1.8	1.2	1.2	1.2	1.5	1.5
^1H swf-tppm decoupling [kHz]	80	80	80	80	80	67	85
Relaxation delay [s]	3.6	3.6	3.2	3.6	3.2	3.5	2.5
Scans	16	16	16	16	16	128	128
SW (f_1/f_2) [kHz]	39/100	39/100	39/100	39/100	39/100	38/100	38/100

Table S2. Experimental parameters of CHHC experiments.

Experiment number	8	9
Probe	Efree	Efree
Carbon isotopic labelling	[1,3-^{13}C]-glycerol	[1,3-^{13}C]-glycerol
^1H Frequency [MHz]	599.8	599.8
MAS rate (ω_r) [kHz]	13	13
Set temperature [℃]	-10.0	-10.0
Carrier frequency [ppm]	103.4	103.4
Mixing time [μs]	150	300
Acquisition points(t_1/t_2)	620/4990	620/4990
Acquisition time (t_1/t_2) [ms]	8.06/24.95	8.06/24.95
Acquisition mode	States	States
$H90[\mu s]$	2.9	2.9
$C90[\mu s]$	5.2	5.2
CP rf power (v_H/v_C) [kHz]	63/48	63/48
First CP contact time [μs]	1500	1500
Second CP contact time [μs]	100	100
Third CP contact time [μs]	100	100
^1H decoupling [kHz]	85	85
Relaxation delay [s]	2.5	2.5
Scans	256	256
SW (f_1/f_2) [kHz]	38/100	38/100

Table S3. Experimental parameters of $CORD_{XY4}$ experiments.

Experiment number	10	11
Probe	Efree	Efree
Carbon isotopic labelling	[1,3-^{13}C]-glycerol	[1,3-^{13}C]-glycerol
^1H Frequency [MHz]	599.8	599.8
MAS rate (ω_r) [kHz]	13	13
Set temperature [℃]	-10.0	-10.0
Carrier frequency [ppm]	100.0	100.0
Mixing time [ms]	150	300
Acquisition points(t_1/t_2)	1450/5988	1024/5988
Acquisition time (t_1/t_2) [ms]	18.58/29.94	13.13/29.94
Acquisition mode	States	States
$H90[\mu s]$	2.6	2.6
$C90[\mu s]$	5.0	5.0
CP rf power (v_H/v_C) [kHz]	63/50	63/50
CP contact time [ms]	1.2	1.2
^1H decoupling [kHz]	75	75
Relaxation delay [s]	3.5	3.2
Scans	32	32
SW (f_1/f_2) [kHz]	39/100	39/100

Processing parameters

Table S4. Apodization details for each of the spectra subsequently used for peak-picking.

Spectrum index	SNR of lowest peak used for peak picking[a]	MAS rate [KHz]	Sample	Experiment	Mixing time [ms]	Apodization functions	
						Direct (F_2)	Indirect (F_1)
1	9	13	Fully	DARR	15	Squared cosine bell	Squared cosine bell
2						Squared cosine bell	200[Hz] exponential
3						100[Hz] exponential	200[Hz] exponential
4						200[Hz] exponential	200[Hz] exponential
5	9	13	Fully	DARR	100	Squared cosine bell	Squared cosine bell
6						Squared cosine bell	200[Hz] exponential
7						100[Hz] exponential	200[Hz] exponential
8						200[Hz] exponential	200[Hz] exponential
9	9	13	Sparse	DARR	50	Squared cosine bell	Squared cosine bell
10						Squared cosine bell	200[Hz] exponential
11						100[Hz] exponential	200[Hz] exponential
12						200[Hz] exponential	200[Hz] exponential
13	9	13	Sparse	DARR	100	Squared cosine bell	Squared cosine bell
14						Squared cosine bell	200[Hz] exponential
15						100[Hz] exponential	200[Hz] exponential
16						200[Hz] exponential	200[Hz] exponential
17	9	13	Sparse	DARR	150	Squared cosine bell	Squared cosine bell
18						Squared cosine bell	200[Hz] exponential
19						100[Hz] exponential	200[Hz] exponential
20						200[Hz] exponential	200[Hz] exponential
21	9	14.5	Sparse	CORD	150	Squared cosine bell	Squared cosine bell
22						Squared cosine bell	200[Hz] exponential

23						100[Hz] exponential	200[Hz] exponential
24						200[Hz] exponential	200[Hz] exponential
25	9	13	Sparse	DARR	300	Squared cosine bell	Squared cosine bell
26						Squared cosine bell	200[Hz] exponential
27						100[Hz] exponential	200[Hz] exponential
28						200[Hz] exponential	200[Hz] exponential
29	9	14	Sparse	CORD	300	Squared cosine bell	Squared cosine bell
30						Squared cosine bell	200[Hz] exponential
31						100[Hz] exponential	200[Hz] exponential
32						200[Hz] exponential	200[Hz] exponential
33	5	13	Sparse	DARR	300	Squared cosine bell	Squared cosine bell
34						Squared cosine bell	200[Hz] exponential
35						100[Hz] exponential	200[Hz] exponential
36						200[Hz] exponential	200[Hz] exponential
37	7	13	Sparse	CHHC	0.15	Squared cosine bell	Squared cosine bell
38	7	13	Sparse	CHHC	0.3	Squared cosine bell	Squared cosine bell

[a]Automated peak-picking was conducted after adjustment of the spectral contour levels. Based on a noise-estimate, the minimal contour level in each spectrum (column 2) was adjusted to a multiple of the signal-to-noise ratio.

Generation of peak lists

Contour level adjustment

Analysis was conducted with NMRFAM-Sparky[9]. For each experiment, the noise level, σ, was estimated by the software according to 5,000 randomly selected points in the 2D spectrum. Next, the first contour level was adjusted to a multiple of σ (i.e., when the minimal contour level was chosen to be 5σ, then only signals at spectral coordinates with a signal-to-noise ratio of 5 or higher would be considered as potential peaks by the automated peak picker). This contour adjustment was conducted manually for each experimental spectrum, in order to enable detection of a maximal number of cross-peaks while avoiding picking a large amount of noise as cross-peaks (minimal contour levels in the range of 5σ-9σ were used, see Table S4).

Automated peak-picking

We used the automated peak-picking feature implemented in NMRFAM-Sparky in order to generate a cross-peak list from the entire spectral range. We defined a minimal drop-off factor of 0.1, that is, two peaks are only considered distinct if the data height drops off by more than 10% in both dimensions before rising again. This prevented excessive peak recognition at overlapping regions of the spectrum. An example of the automated peak-picking process is visualized in Fig. S1.

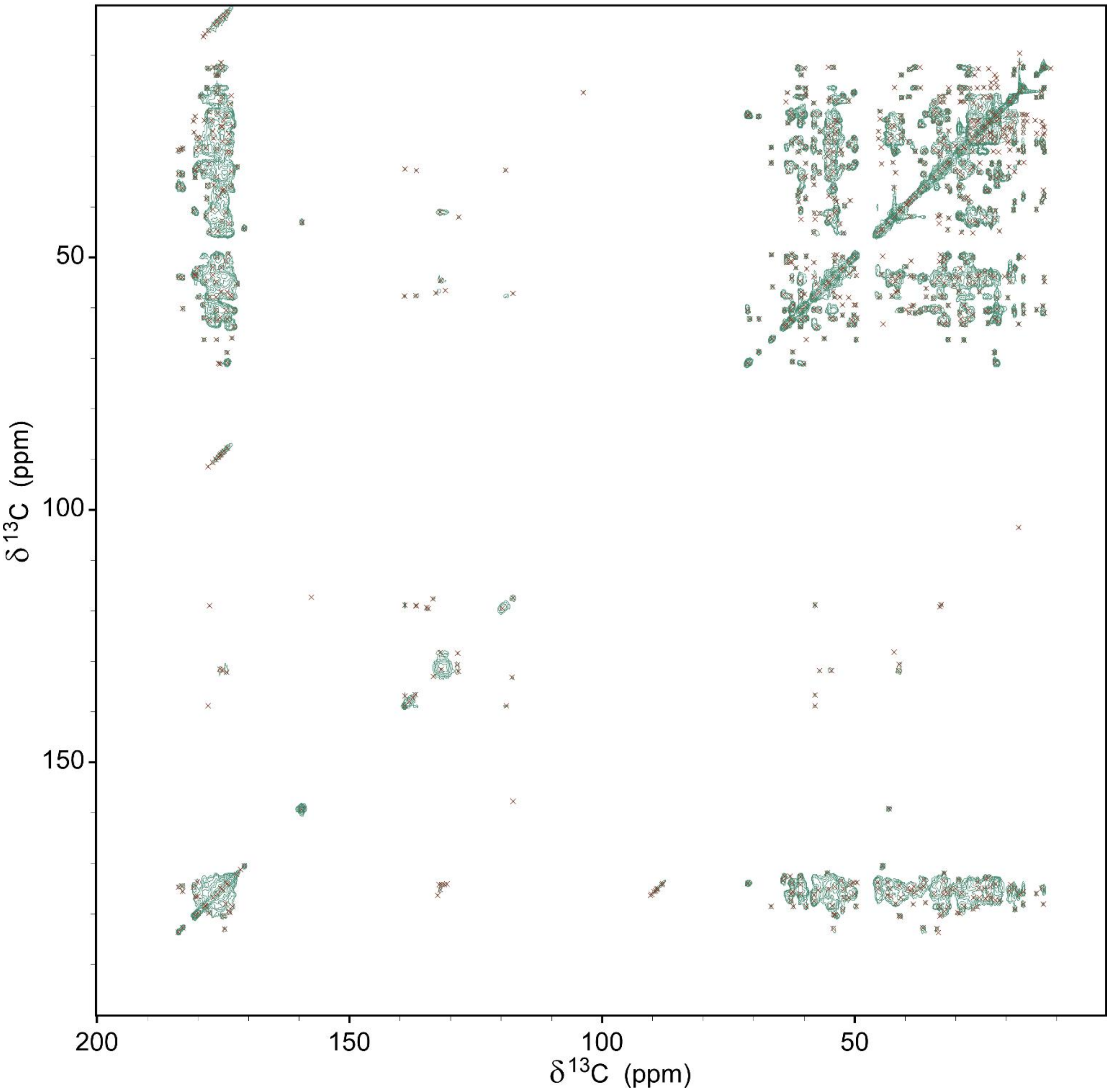

Figure S1. DARR spectrum of U-^{13}C-labeled ssDNA-bound gVp, acquired with a mixing time of 100[ms]. 14 contour levels are plotted at multiples of 1.4 with the lowest contour level adjusted to an SNR value of 9. Peaks detected by the automated peak-picking tool of Sparky are marked with ‘x’. Both dimensions were apodized by a squared cosine function.

Filtration of spinning-sidebands and diagonal peaks

A home-built python script was used to filter out cross-peaks belonging to spinning sidebands and diagonal cross-peaks from the peak lists. An example of the use of the peak filtration code is shown in Fig. S2.

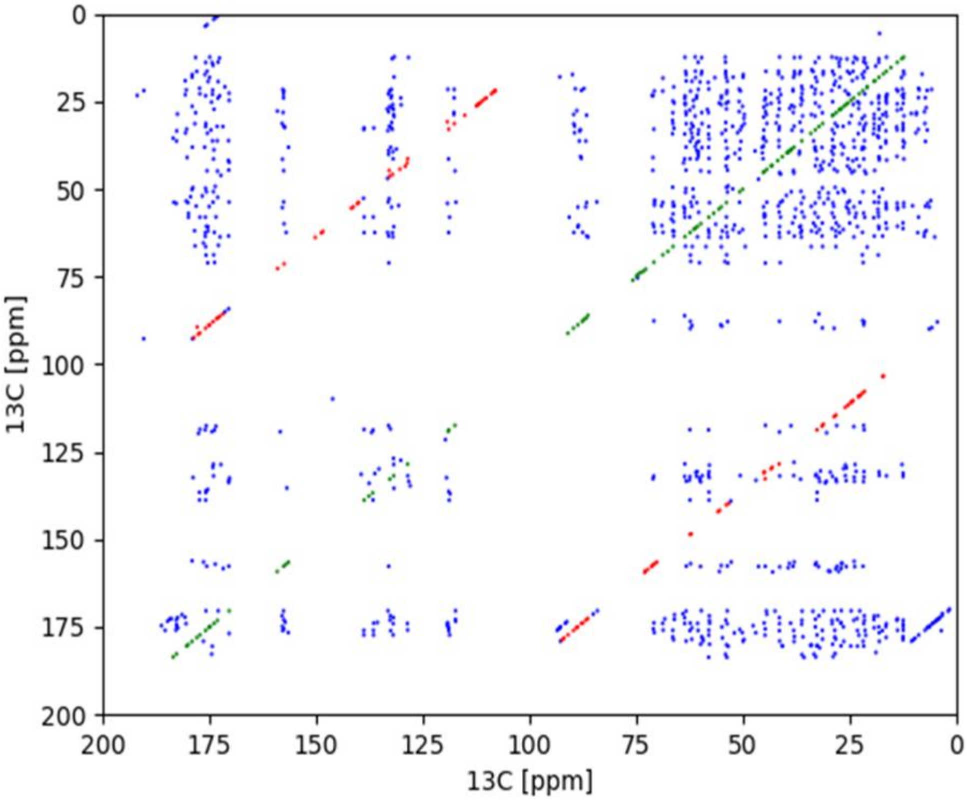

Figure S2. Each data point represents a peak detected by the automated peak-picking process in Sparky, from a DARR spectrum of [1,3-^{13}C]-glycerol-labeled ssDNA-bound gVp, acquired with a mixing time of 300[ms] (Exp 7 In Table S1) and apodized with a squared cosine function in both dimensions. Diagonal peaks detected by the Python script are marked in green, and spinning sidebands are marked in red. Both categories are removed from any further analysis. Blue peaks were subsequently considered for the extraction of distance restraints.

Generation of distance restraints

Initial elucidation of restraints based on peak lists

We produced distance restraints based on the filtered peak lists described above, for both isotopic labeling schemes used – [1,3-^{13}C]-glycerol (gly13) and U-[^{13}C]-glucose (glcU). All peak lists (38 in total, see Table S4) were analyzed with the chosen chemical shift tolerance value of 0.3[ppm].
The input to the restraint analysis of glcU data was a concatenated list of all peak lists from all spectra collected with all apodization methods used, where redundant restraints were merged. A similar process was applied for the data from the gly13 sample. Additionally, we used for this sample a probability threshold of 0.4, meaning that we only considered a correlation as a possible assignment of a cross-peak if the product of the probabilities of both ^{13}C nuclei was 40% and higher. This intermediate value was used in an attempt to maximize the information elucidated from the sparsely labeled sample, while avoiding the risk of ruling out possible assignments corresponding to pairs of carbon sites that may have a low probability of being isotopically labeled due to scrambling but may indeed be the source of a detected spectral correlation. Finally, for each list we kept peaks up to a level of ambiguity of 20.

Aggregation of distance restraints across fully and sparsely labeled samples

We aggregated both sets of distances restraints (glcU and gly13) in order to generate a single set of restraints to be given as input to the calculation process. We gave precedence to the data derived from spectra of the sparsely labeled sample over that derived from spectra of the fully labeled sample, since its ambiguity level is lower on average, resulting in more informative structural constraints. First, we appended all restraints arising from the sparsely labeled data sets to the aggregated list of restraints. Second, we iterated over these sparse restraints; for each one, we removed any restraints arising from fully labeled data corresponding to a cross-peak within a 0.3[ppm] chemical shift tolerance window around the coordinates of the cross-peak associated with the sparse restraint. Finally, we appended all the restraints elucidated from fully labeled data that were not discarded by the above process to the aggregated list of restraints.

Distance restraint filtration

We applied three filters on the aggregated list of restraints described above:

- Removed restraints including single-bond contacts – such contacts result in strong dipolar coupling, therefore we assumed that with sufficiently high probability, the single-bond contact is the source of the detected correlation, as opposed to other longer-range possible assignments. Since single-bond contacts do not report on the overall protein fold, we discarded such restraints.
- Discarded possible assignments with an internuclear distance larger than 16[Å] in the free gVp X-ray structure – we assumed that the overall protein fold is not changed entirely upon binding to ssDNA. Therefore, we could safely discard possible assignments corresponding to pairs of carbon sites that are far apart in the free gVp structure. We used a large cutoff value of 16[Å] in order to reduce the risk of ruling out the true assignment in this process.
- Removed restraints that included a possible assignment with an inter-monomer distance shorter than 7[Å] in the free gVp X-ray homodimer structure – this reduces the risk of erroneously designating inter-monomer correlations as intra-monomer, which would result in false distance restraints for the calculation of the monomer structure.

Structure calculation protocol

Structure calculation was performed using Xplor-NIH[10,11] executed on NMRBox[8].

Initial iteration: 250 structures generated. Top 25 lowest energy structures used for violation analysis as follows: (i) Distance restraints violated by more than 2.0[Å] in at least 50% of the 25 structures were removed. (ii) Angle restraints violated by more than 10° (beyond the error value reported for the angle prediction by TALOS+) in at least 50% of the structures were removed. The updated restraints were used as input to a second run using the same Xplor script.

Second iteration: 250 structures generated. Top 25 lowest energy structures used for violation analysis. Next, a regularized, average structure was calculated based on these 25 structures. All possible assignments corresponding to a pair of carbon nuclei further apart than a cutoff distance of 16[Å] in the average structure were removed.

iterations 3-10: 200 structures were generated using the above mentioned 16[Å] cutoff from the average structure, top 20 lowest energy structures analyzed. Violation analysis performed as above. After iteration 3, we utilized iteratively decreasing cutoff values of 14[Å], 12[Å], 10[Å], 9.5[Å], 9.25[Å], 9[Å], and 8.75[Å] from the average structure for iterations 4-10.

iteration 11: 200 structures generated, top 10 lowest energy structures analyzed, cutoff used 8.5[Å]. Violation analysis conducted with more strict thresholds – angle restraints were ruled out if they were violated in at least 25% of the 10 structures by more than 10°, and distance restraints were ruled out if they were violated in at least 25% of these structures by more than 1.5[Å].

iteration 12: 200 structures generated, top 10 lowest energy structures analyzed, no cutoff used. The top 10 lowest energy structures of this iteration were the output of the calculation process prior to refinement. They were used for the final analysis, in order to generate the final restraints to be provided as input to the refinement step. Strict thresholds were used for filtration by violations – angle restraints were ruled out if they were violated by more than 5° in at least 25% of the 10 structures, and distance restraints were ruled out if they were violated in at least 25% of these structures by more than 1.5[Å].

Final iteration: structure refinement in implicit solvent. Used the very final set of distance and angle restraints from iteration 12, and the lowest energy structure from iteration 12 was used as the initial structure for the refinement process. 100 refined models were calculated and the top 10 lowest energy structures were reported as the final structural ensemble of our research.

For all structure iteration runs with the exclusion of the final refinement step, the “soft” form (soft-square potential) for the corresponding potential energy in Xplor was used, so that erroneously assigned cross-peak would not lead to significant structural distortions. Structure refinement was performed with the “hard” form (square-well potential).

Iteration details

Table S5. Parameter values used throughout the calculation process.

Calculation iteration	1	2	3	4	5	6	7	8	9	10	11	12	13 (refine)
k	25	25	20	20	20	20	20	20	20	20	10	10	10
N	250	250	200	200	200	200	200	200	200	200	200	200	100
Distance restraints violation criterion [Å]	-	2.0	2.0	2.0	2.0	2.0	2.0	2.0	2.0	2.0	2.0	1.5	1.5
Share of k-ensemble for distance restraint violation	-	50%	50%	50%	50%	50%	50%	50%	50%	50%	50%	25%	25%
Number of input distance restraints	1901	1825	1786	1741	1661	1506	1409	1382	1321	1288	1253	1248	1247
Angle restraints violation criterion[°]	-	10	10	10	10	10	10	10	10	10	10	10	5
Share of k-ensemble for angle restraint violation	-	50%	50%	50%	50%	50%	50%	50%	50%	50%	50%	25%	25%
Number of input angle restraints	142	126	125	125	125	125	124	123	122	122	122	121	112
Potential function of distance restraints	soft	soft	soft	soft	soft	soft	soft	soft	soft	soft	soft	soft	hard
Cutoff from average structure [Å]	-	-	16.0	14.0	12.0	10.0	9.5	9.25	9.0	8.75	8.5	-	-
k-ensemble Cα-RMSD [Å]	5.17	5.48	5.76	5.48	4.71	3.78	2.92	3.37	3.26	3.00	2.38	2.55	1.20
k-ensemble Average energy scoring [kcal/mol]	9525	3297	3120	2827	3136	2540	1769	1659	1576	1146	926	728	586

Xplor-NIH scripts

Structure generation script

```
#coding: utf-8
import protocol
protocol.initStruct("gvp_sequence.psf")
protocol.initParams("protein")
protocol.initRandomSeed(3421)
protocol.genExtendedStructure()
from potList import PotList
etotal = PotList()
from simulationTools import MultRamp, StaticRamp, InitialParams
highTempParams = []
rampedParams = []
import noePotTools
noe = noePotTools.create_NOEPot(name="noe", file="distnace_restraints.tbl")
noe.setPotType("soft")
etotal.append(noe)
rampedParams.append(MultRamp(2, 30, "noe.setScale(VALUE)"))
from xplorPot import XplorPot
dihedralTable = "angle_restraints.tbl"
protocol.initDihedrals(dihedralTable)
etotal.append(XplorPot("CDIH"))
highTempParams.append(StaticRamp("etotal['CDIH'].setScale(10)"))
rampedParams.append(StaticRamp("etotal['CDIH'].setScale(200)"))
import hbPotTools
hbond = hbPotTools.create_HBPot('hbond')
hbond.setScale(2.5)
etotal.append(hbond)
import torsionDBPotTools
torsiondb = torsionDBPotTools.create_TorsionDBPot(name="torsiondb",
system="protein")
etotal.append(torsiondb)
rampedParams.append(MultRamp(0.002, 2, "torsiondb.setScale(VALUE)"))
from repelPotTools import create_RepelPot, initRepel
repel = create_RepelPot("repel")
etotal.append(repel)
highTempParams.append( StaticRamp("""initRepel(repel,
use14=True,
scale=0.004,
repel=1.2,
moveTol=45,
#interactingAtoms=’name CA’
interactingAtoms='name CA'
)""") )
rampedParams.append(StaticRamp("initRepel(repel, use14=False)"))
rampedParams.append(MultRamp(0.004, 4, "repel.setScale(VALUE)"))
import torsionDBPotTools
repel14 = torsionDBPotTools.create_Terminal14Pot("repel14")
etotal.append(repel14)
highTempParams.append(StaticRamp("repel14.setScale(0)"))
rampedParams.append(MultRamp(0.004, 4, "repel14.setScale(VALUE)"))
etotal.append(XplorPot("BOND"))
etotal.append(XplorPot("ANGL"))
rampedParams.append(MultRamp(0.4, 1.0, "etotal['ANGL'].setScale(VALUE)"))
etotal.append(XplorPot("IMPR"))
rampedParams.append(MultRamp(0.1, 1.0, "etotal['IMPR'].setScale(VALUE)"))
from ivm import IVM
dyn = IVM()
protocol.torsionTopology(dyn)
minc = IVM()
protocol.cartesianTopology(minc)
protocol.massSetup()
temp_ini = 3500.0
temp_fin = 25.0
def calcOneStructure(loopInfo):
```

```
    """Calculate a single structure."""
    from monteCarlo import randomizeTorsions
    randomizeTorsions(dyn)
    import torsionTools
    torsionTools.setTorsionsFromTable(dihedralTable)
    protocol.fixupCovalentGeom(maxIters=100, useVDW=True)
    InitialParams(rampedParams)
    InitialParams(highTempParams)
    protocol.initDynamics(dyn,
    potList=etotal,
    bathTemp=temp_ini,
    initVelocities=True,
    finalTime=100,
    numSteps=1000,
    printInterval=100)

    dyn.setETolerance(temp_ini/100)
    dyn.run()

    protocol.initDynamics(dyn,
    finalTime=0.2,
    numSteps=100,
    printInterval=100)

    from simulationTools import AnnealIVM
    AnnealIVM(initTemp=temp_ini,
    finalTemp=temp_fin,
    tempStep=12.5,
    ivm=dyn,
    rampedParams=rampedParams).run()
    protocol.initMinimize(dyn,
    printInterval=50)
    dyn.run()

    protocol.initMinimize(minc,
    potList=etotal,
    dEPred=10)
    minc.run()

from simulationTools import StructureLoop
StructureLoop(numStructures=200,
structLoopAction=calcOneStructure,
doWriteStructures=True,
genViolationStats=True,
averageSortPots=[etotal["BOND"], etotal["ANGL"], etotal["IMPR"], noe, etotal["CDIH"]],
averageTopFraction=0.1,
averagePotList=etotal,
averageFitSel="name CA",
averageFilename="SCRIPT_ave.pdb",
).run()
```

Refinement script

```
#coding: utf-8
import protocol

opts,args = xplor.parseArguments(["quick:0"])
quick=True if opts and opts[0][0] == "quick" else False

numberOfStructures= 100

inTemplate="fold" + "_*.pdb"
outfilename = 'SCRIPT_STRUCTURE.sa'

protocol.initRandomSeed(3421)

import eefxPotTools
eefxPotTools.initEEFx()

import glob
protocol.loadPDB( glob.glob(inTemplate)[0],
                  deleteUnknownAtoms=True )


from potList import PotList
potList = PotList()
crossTerms = PotList('cross')

from simulationTools import MultRamp, StaticRamp
hiTempParams = []
rampedParams = []

from noePotTools import create_NOEPot
noe = create_NOEPot('noe', 'distance_restraints.tbl')
noe.setPotType("hard")
potList.append(noe)
rampedParams.append(MultRamp(2.0,30.0, "noe.setScale( VALUE )"))

torsionFile='angle_restraints.tbl'
protocol.initDihedrals(torsionFile)
from xplorPot import XplorPot
potList.append(XplorPot('CDIH'))
hiTempParams.append( StaticRamp("potList['CDIH'].setScale(10)") )
rampedParams.append( StaticRamp("potList['CDIH'].setScale(200)") )

from torsionDBPotTools import create_TorsionDBPot
torsionDBPot = create_TorsionDBPot('tDB')
potList.append( torsionDBPot )
rampedParams.append( MultRamp(.002,2,"torsionDBPot.setScale(VALUE)") )

import hbPotTools
hbond = hbPotTools.create_HBPot('hbond')
hbond.setScale(2.5)
potList.append(hbond)

from xplorPot import XplorPot
for term in ('BOND', 'ANGL', 'IMPR'):
    potList.append( XplorPot(term) )
    pass

potList['ANGL'].setThreshold(5.0)
potList['IMPR'].setThreshold(5.0)

rampedParams.append(MultRamp(0.4, 1.0, "potList['ANGL'].setScale(VALUE)"))
rampedParams.append(MultRamp(0.1, 1.0, "potList['IMPR'].setScale(VALUE)"))

from eefxPotTools import create_EEFxPot, param_LK
eefxpot=create_EEFxPot("eefxpot")
eefxpot.setVerbose(False)
```

```
potList.append(eefxpot)
rampedParams.append(MultRamp(0.1,1.0,"eefxpot.setScale(VALUE)"))

from ivm import IVM
dyn = IVM()
protocol.torsionTopology(dyn)

minc = IVM()
protocol.cartesianTopology(minc)

temp_ini = 3500.0
temp_fin = 25.0

protocol.massSetup()

def calcOneStructure(loopInfo):
    from simulationTools import InitialParams
    InitialParams(rampedParams)
    InitialParams(hiTempParams)

    protocol.initDynamics(dyn,
                          potList=potList,
                          bathTemp=temp_ini,
                          initVelocities=True,
                          finalTime=15,
                          numSteps=50 if quick else 15001,
                          printInterval=100)

    dyn.setETolerance(temp_ini/100)
    dyn.run()

    InitialParams(rampedParams)

    protocol.initDynamics(dyn,
                          potList=potList,
                          finalTime=0.4,
                          numSteps=2 if quick else 201,
                          printInterval=100)

    from simulationTools import AnnealIVM
    AnnealIVM(initTemp=temp_ini,
              finalTemp=temp_fin,
              tempStep=1000 if quick else 12.5,
              ivm=dyn,
              rampedParams=rampedParams).run()

    protocol.initMinimize(dyn,
                          potList=potList,
                          printInterval=50)
    if not quick: dyn.run()

    protocol.initMinimize(minc,
                          potList=potList,
                          dEPred=10)
    if not quick: minc.run()
    return

from simulationTools import FinalParams, StructureLoop
StructureLoop(numStructures=numberOfStructures,
              doWriteStructures=True,
              averagePotList=potList,
              averageContext=FinalParams(rampedParams),
              pdbTemplate=outfilename,
              structLoopAction=calcOneStructure,
              genViolationStats=True,
              averageSortPots=[potList["BOND"],potList["ANGL"],potList["IMPR"],noe,potList["CDIH"]],
              averageTopFraction=0.1,
              averageFilename="SCRIPT_ave.pdb",
              ).run()
```

Restraints input to refinement step

Depicted below are the number of distance and angles restraints provided as input to the refinement step, along with the number of restraints that are violated by the final 10-structure ensemble. Also, the number of unambiguous distance restraints provided as input to the final refinement step are specified according to their type of contact. A distance restraint is defined as violated if the effective distance deviates by more than 0.5[Å] from the 2-8[Å] range enforced, and angle restraint is defined as violated if the angle deviates by more than 5° beyond the error margins of the value of the angle predicted via TALOS+.

Table S6. Distance and angle restraint input to refinement step.

Restraints	Input	Violations
Distance	1247	15
Torsion angle	112	7

Table S7. Unambiguous distance restraint input to refinement step.

Restraint type	Number of restraints
Intra-residue	80
Sequential	112
Medium-range	126
Long-range	275

Violation analysis in the refined ensemble

Described below are the distance and angle restraints violated (by more than 0.5[Å] or 5°, respectively) in some or all members of the final 10-structure ensemble deposited to the Protein Data Bank.

Table S8. Violations of torsion angle restraints.

Type of torsion angle	Atomic definition of the torsion angle	Violation size[°]	Share of ensemble members in violation of the restraint
ϕ	L65C-S66N-S66Cα-S66C	6.08	40%
ψ	T62N-T62Cα-T62C-V63N	5.75	20%
ψ	R16N-R16Cα-R16C-S17N	5.92	10%
ϕ	R16C-S17N-S17Cα-S17C	5.92	10%
ψ	N39N-N39Cα-N39C-E40N	6.09	10%
ψ	L49N-L49Cα-L49C-D50N	5.32	10%
ψ	Y56N-Y56Cα-Y56C-A57N	5.53	10%

Table S9. Violations of distance angle restraints.

Ambiguity level	restraint	Violation size[Å]	Share of ensemble members in violation of the restraint
1 (long-range)	[34Cε2-45C]	0.81	100%
1 (long-range)	[45Cγ1-5Cγ]	0.56	60%
1 (long-range)	[53Cγ-79Cβ]	0.63	30%
1 (long-range)	[3C-47Cα]	0.61	20%
1 (long-range)	[67Cβ-76Cα]	0.58	20%
1 (medium-range)	[18C-21Cβ]	0.51	20$
1 (long-range)	[34Cβ-61Cβ]	0.59	20%
1 (long-range)	[29Cα-48Cα]	0.7	10%
1 (long-range)	[30Cγ-53Cγ]	0.51	10%
1 (medium-range)	[51Cα-54Cα]	0.58	10%
1 (long-range)	[29Cα-50Cα]	0.58	10%
1 (long-range)	[4Cγ2-86Cβ]	0.54	10%
1 (sequential)	[40Cβ-41Cε2]	0.5	10%
1 (long-range)	[68Cδ1-78Cγ2]	0.59	10%
1 (medium-range)	[65Cα-68Cβ]	0.67	10%

Distribution of distance restraints by ambiguity level throughout the calculation process

Depicted below, for each iteration of the calculation process, is the distribution of distance restraints according to the ambiguity level. As can be seen, iteratively the restraints become less ambiguous on average.

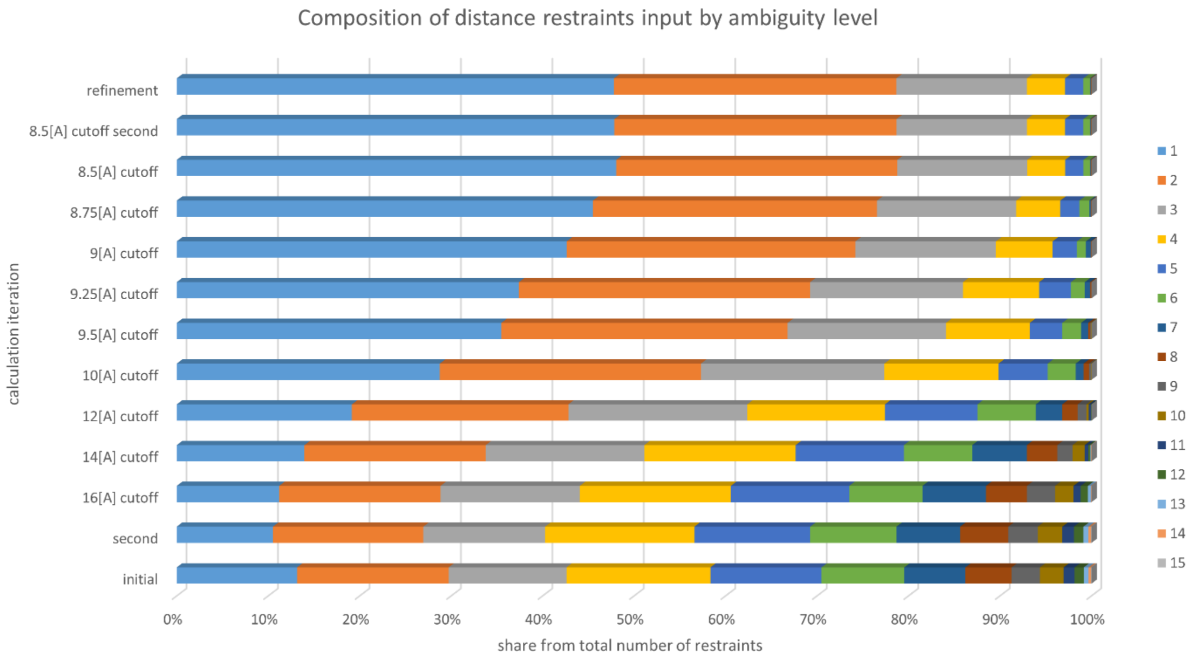

Figure S3. Distribution of distance restraints provided as input to each of the thirteen calculation steps according to the level of ambiguity (the number of possible assignments included in the restraints).

Cα-RMSD and energy score throughout the calculation process

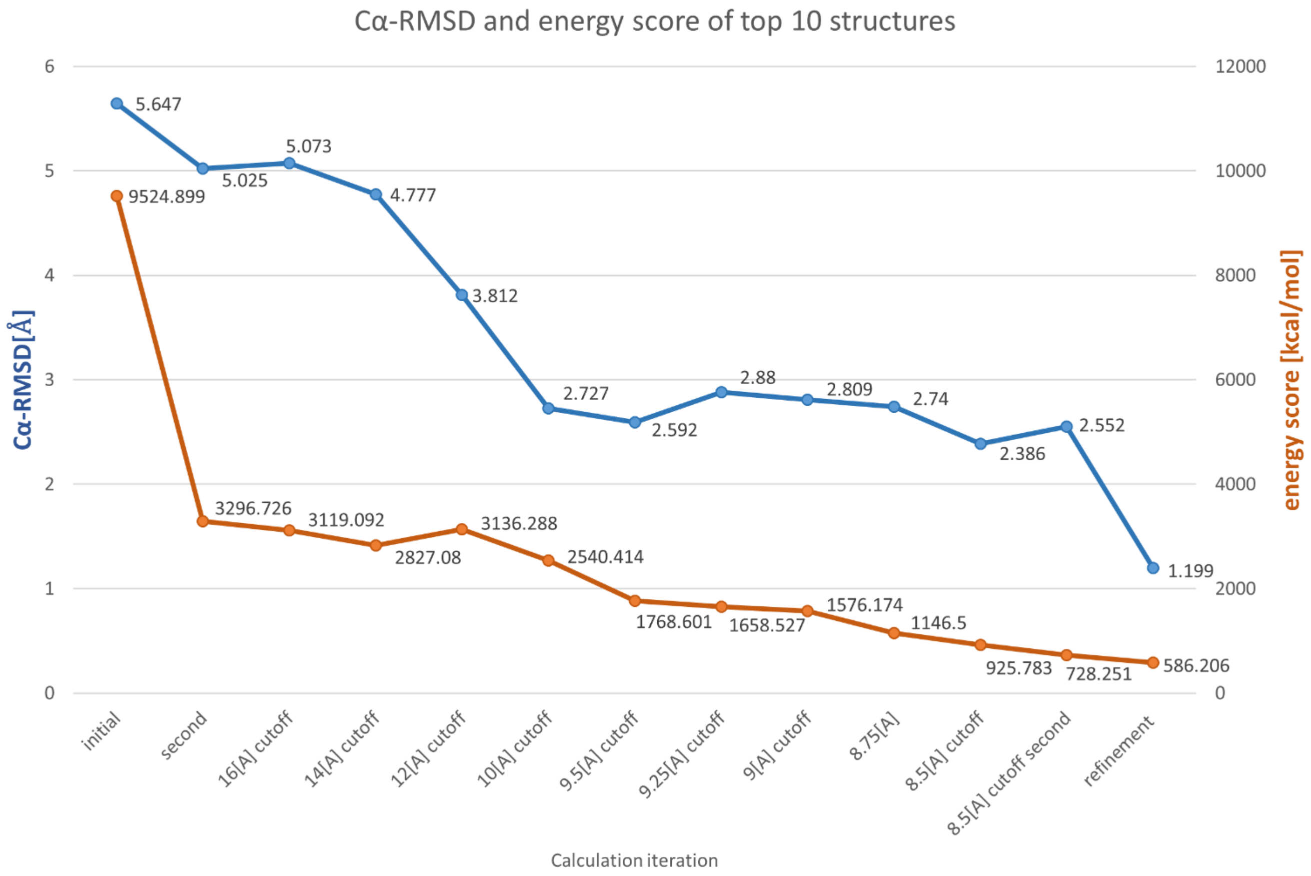

Figure S4. Progression of the average sorting energy score of the top 10 lowest energy structures throughout the calculation process (orange), and progression of the $C\alpha$-RMSD of the 10 structures against their average structure (blue).

Ensemble validation

Ramachandran plot of torsion angles

The Ramachandran plot of our refined 10-structure ensemble is depicted in Fig. S5 (generated by PSVS v.15[12] and PROCHECK v3.5.[13]), with the corresponding analysis given in Table S3. Out of the 87 residues in gVp, 57 were deemed ordered by PSVS in our ensemble according to their dihedral angle order parameters (well-ordered residues of the ensemble: 3-13, 15-17, 27-30, 33-36, 41-49, 53-62, 68-77, 81-86). Out of these 57 residues, 3 are glycine residues and 5 are proline residues. These 8 residues are excluded from the analysis. Therefore, the plot depicts 490 data points (49 residues in 10 structures). 100% of the analyzed residues reside at the most favored or additional allowed regions, and none of the 490 analyzed residues have ϕ-ψ coordinates in the disallowed region. This is a strong indication of the validity of the structure, and this means that most of the torsion angles describing the overall backbone conformation are not sterically improbable due to steric clashes. The entire Ramachandran statistics are provided in Table S10.

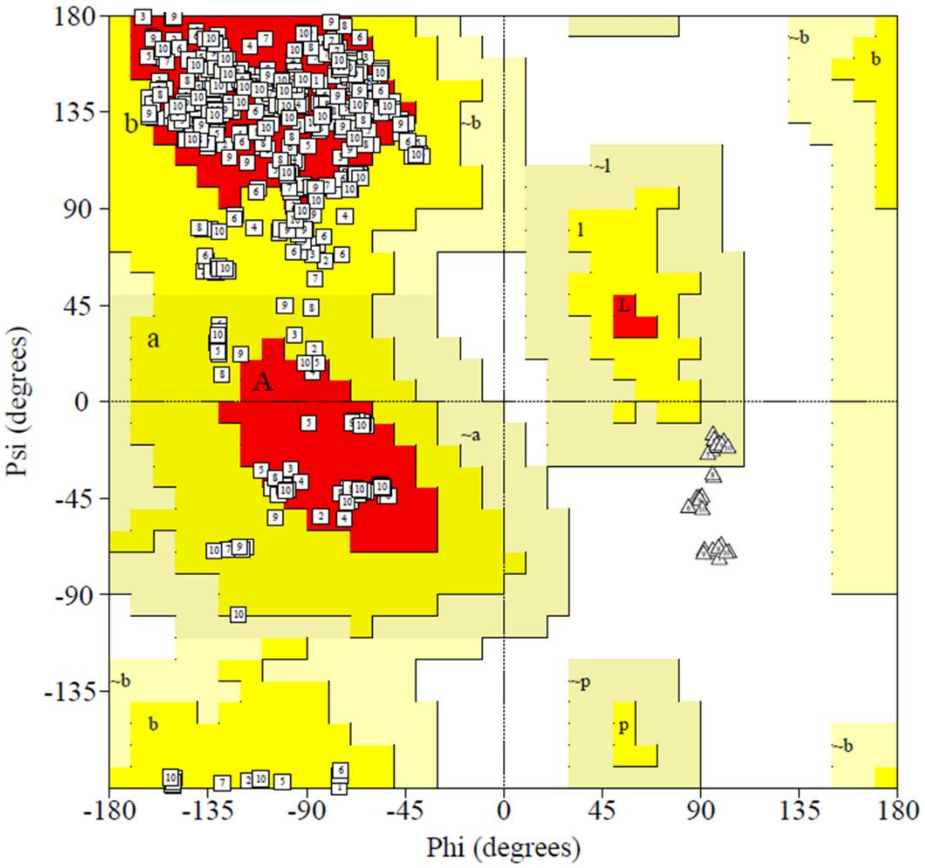

Figure S5. Ramachandran plot for all 10 members of the refined ensemble (generated via PSVS and PROCHECK). Glycine residues are shown as triangles, and other residue types are shown as squares.

Table S10. Categorization of the 500 data points included in the Ramachandran plot according to the four different probabilistic regions defined by PROCHECK (based on a calculation of the number of residues that are located within each 10°x10° pixels, out of a databank of 121,870 residues from 463 structures). The most favoured (A, B, L in Fig. S5) and additional allowed regions (a, b, l, p) correspond to pixels with more than 100 and 8 residues, respectively. The generously allowed regions (~a, ~b, ~l, ~p) are defined by an extension of two pixels around the additional allowed regions. All other regions of the 2D ϕ-ψ space are considered disallowed.

Plot region	Number of residues	Percentage from all analysed residues
Most favoured [A,B,L]	380	77.6%
Additional allowed [a,b,l,p]	110	22.4%
Generously allowed [~a, ~b, ~l, ~p]	0	0.0%
Disallowed	0	0.0%

Ramachandran plot comparison of free and bound gVp

Torsion angles in the Ramachandran space corresponding to the same set of atoms in the X-ray structure of free gVp (circles) and in the ssNMR structure of the bound form (squares) are connected by a line. When the secondary structure in the bound and free forms is retained (according to Stride) the torsion angle data points are filled. Circles corresponding alpha-helix regions in the free gVp are plotted in blue for clarity. While none of alpha-helix residues of free gVp are detected in the reported model, it can be seen that this is mainly due to slight changes in ψ-ϕ space upon binding. Most of the residues not determined as beta-strands in the bound form have torsion angles that undergo significant changes at least in part of the stretch thus preventing the region from being recognized as a beta strand. Glycine and proline residues are excluded from the plot.

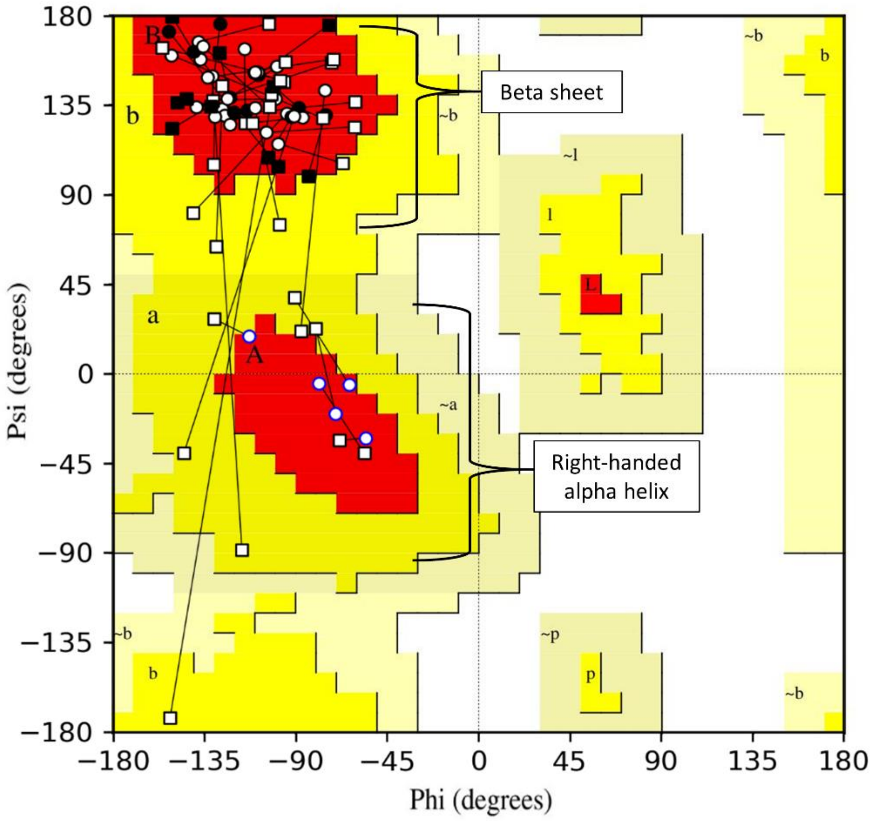

Figure S6. Ramachandran plot comparison of secondary structure regions in free and ssDNA-bound gVp.

MolProbity clashscore

MolProbity is a tool widely used for structural validation of protein and nucleic acids models[14]. The software conducts all-atom contact analysis in order to detect local structural errors in the three-dimensional model. The MolProbity clash score, which is implemented as one the main structure quality criteria reported for structures in the protein data bank, is the number of inter-atomic steric clash overlaps (between non-bonded atoms) larger than 0.4[Å] per 1,000 atoms[15,16], suggesting that at least one of the two atoms involved is modeled incorrectly. The 0.4[Å] threshold is intended to balance the diagnosis of conformational errors with the raising of false alarms.
We used the ONEDEP software of the worldwide protein data bank (wwPDB) in order to run MolProbity on our refined ensemble. This software does not count clashes that include at least one atom in an ill-defined region of the protein in the ensemble of structures (regions deemed in well-defined by the software for our ensemble includes residues 2-16, 28-37 and 41-87, with a total of 72 out 87 residues; the ill-defined region is mainly composed of the DNA-binding loop). The calculated MolProbity clash score is 26, which is similar to values reported by the PDB for other protein structures elucidated via MAS ssNMR (CAP-Gly bound to microtubules – PDB ID: 2MPX[17] – 24; Crh dimer – PDB ID: 2RLZ[18] – 27; GB1 – PDB ID: 2KQ4[19] – 28).

Unambiguous long-range restraints at regions of interest

Location of C-terminus with respect to the core

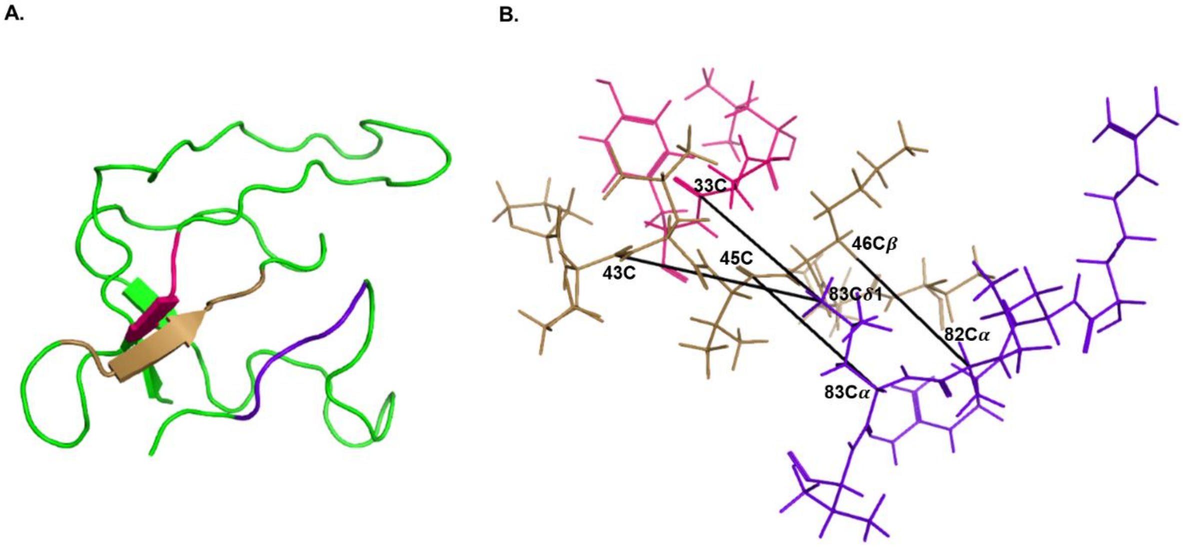

Figure S7. Visualization of unambiguous long-range restraints justifying the location of the C-terminus with respect to the core as well as the formation of two beta strands. A. The ensemble-average structure calculated for bound gVp, with regions of interest coloured (pink – residues 32-34, olive yellow – residues 42-48, purple – residues 80-84). B. The four correlations are depicted as black lines.

Table S11. Unambiguous restraints constraining the location of the C-terminus with respect to the core.

Restraint	Distance
33C – 83Cδ1	7.0[Å]
43C – 83Cδ1	7.9[Å]
45C – 83Cα	7.0[Å]
46Cβ – 82Cα	7.3[Å]

Proximity of dyad loop and core loop

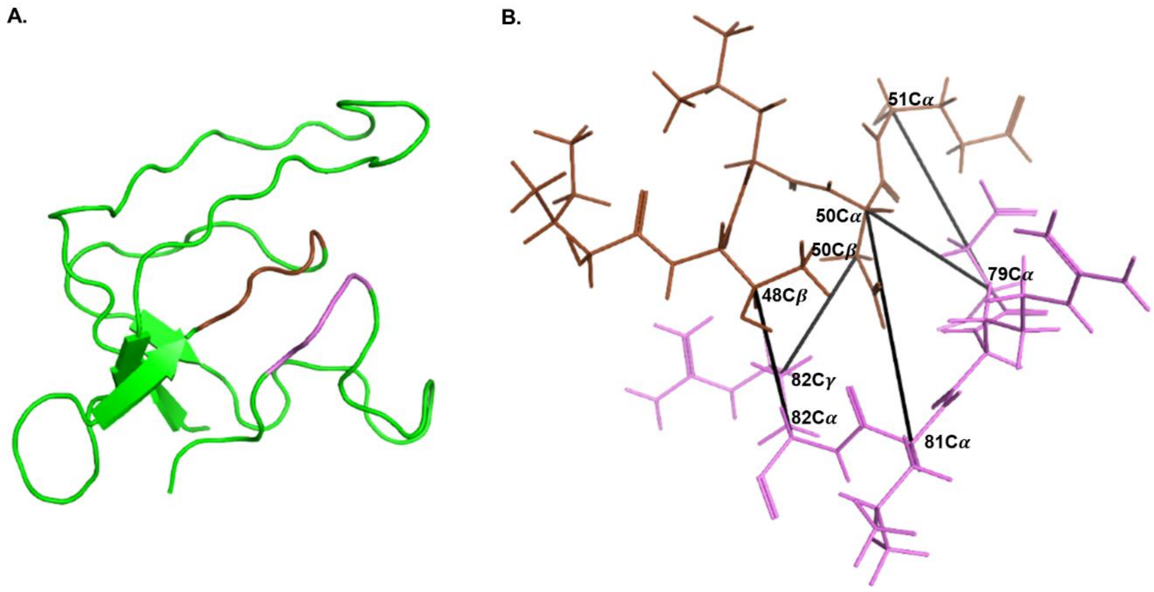

Figure S8. Visualization of unambiguous long-range restraints justifying the proximity of the dyad loop, as well as subsequent residues in the sequence, to the core loop. A. The ensemble-average structure calculated for bound gVp, with regions of interest coloured (brown – residues 47-53, red – residues 79-82). B. The four correlations are depicted as black lines.

Table S12. Unambiguous restraints constraining the location of the dyad loop with respect to the core loop.

Restraint	Distance
50Cα – 79Cα	4.7 [$\AA$]
50Cα – 81Cα	8.0[$\AA$]
50Cβ – 82Cγ	4.5[$\AA$]
51Cα – 79Cα	6.6[$\AA$]
48Cβ – 82Cα	4.6[$\AA$]

Inter-loop distances

Table S13. Inter-loop distances for free and ssDNA-bound gVp structures. Each inter-loop distance is calculated as the average of all pairwise Cα-Cα inter-loop distances

Inter-loop distance [Å]	DNA-binding-loop – core-loop	Dyad-loop – core-loop	DNA-binding-loop – dyad-loop
Free X-ray structure	18.4	25.2	26.0
ssDNA-bound ensemble members (ssNMR)			
1	14.2	18.3	21.5
2	14.1	18.4	22.0
3	13.7	18.0	19.4
4	14.3	18.5	21.9
5	14.1	19.3	22.2
6	13.9	19.2	21.8
7	13.7	18.3	21.2
8	14.0	18.3	21.3
9	13.6	18.9	22.0
10	14.3	19.0	22.6
Ensemble average [Å]	14.0	18.6	21.6
Standard deviation [Å]	1.35	2.03	1.57
**Δ**(free-bound) [Å]	4.41	6.58	4.41

References

(1) Kedem, S.; Hassid, R. R.; Shamir, Y.; Goldbourt, A. Conformational Changes of Ff Phage Protein GVp upon Complexation with Its Viral SsDNA : Evidence from Magic-Angle-Spinning Solid-State NMR. *Viruses* **2022**, *14* (6), 1264.

(2) Hassid, R. R.; Kedem, S.; Bachar-Beck, M.; Shamir, Y.; Goldbourt, A. Solid State NMR Chemical Shift Assignment of the Non-Structural Single-Stranded DNA Binding Protein GVp from Fd Bacteriophage. *Biomol. NMR Assign.* **2022**.

(3) Stassen, A. P. M.; Folmer, R. H. A.; Hilbers, C. W.; Konings, R. N. H. Single-Stranded DNA Binding Protein Encoded by the Filamentous Bacteriophage M13: Structural and Functional Characteristics. *Mol. Biol. Rep.* **1994**, *20* (3), 109–127.

(4) Takegoshi, K., S. Nakamura, T. T. 13C-1H Dipolar-Assisted Rotational Resonance in Magic-Angle Spinning NMR. *Chem. Phys. Lett.* **2001**, *344* (5–6), 631–637.

(5) Hou, G.; Yan, S.; Trébosc, J.; Amoureux, J. P.; Polenova, T. Broadband Homonuclear Correlation Spectroscopy Driven by Combined R2 vn Sequences under Fast Magic Angle Spinning for NMR Structural Analysis of Organic and Biological Solids. *J. Magn. Reson.* **2013**, *232*, 18–30.

(6) Wilhelm, M.; Feng, H.; Tracht, U.; Spiess, H. W. 2D CP/MAS 13C Isotropic Chemical Shift Correlation Established by 1H Spin Diffusion. *J. Magn. Reson.* **1998**, *134* (2), 255–260.

(7) Delaglio, F.; Grzesiek, S.; Vuister, G. W.; Zhu, G.; Pfeifer, J.; Bax, A. NMRPipe: A Multidimensional Spectral Processing System Based on UNIX Pipes. *J. Biomol. NMR* **1995**, *6* (3), 277–293.

(8) Maciejewski, M. W.; Schuyler, A. D.; Gryk, M. R.; Moraru, I. I.; Romero, P. R.; Ulrich, E. L.; Eghbalnia, H. R.; Livny, M.; Delaglio, F.; Hoch, J. C. NMRbox: A Resource for Biomolecular NMR Computation. *Biophys. J.* **2017**, *112* (8), 1529–1534.

(9) Lee, W.; Tonelli, M.; Markley, J. L. NMRFAM-SPARKY: Enhanced Software for Biomolecular NMR Spectroscopy. *Bioinformatics* **2015**, *31* (8), 1325–1327.

(10) Schwieters, C. D.; Kuszewski, J. J.; Tjandra, N.; Clore, G. M. The Xplor-NIH NMR Molecular Structure Determination Package. *J. Magn. Reson.* **2003**, *160* (1), 65–73.

(11) Schwieters, C. D.; Kuszewski, J. J.; Marius Clore, G. Using Xplor-NIH for NMR Molecular Structure Determination. *Prog. Nucl. Magn. Reson. Spectrosc.* **2006**, *48* (1), 47–62.

(12) Bhattacharya, A.; Tejero, R.; Montelione, G. T. Evaluating Protein Structures Determined by Structural Genomics Consortia. *Proteins* **2007**, *66* (4), 778–795.

(13) Laskowski, R. A.; MacArthur, M. W.; Moss, D. S.; Thornton, J. M. PROCHECK: A Program to Check the Stereochemical Quality of Protein Structures. *J. Appl. Crystallogr.* **1993**, *26* (2), 283–291.

(14) Williams, C. J.; Headd, J. J.; Moriarty, N. W.; Prisant, M. G.; Videau, L. L.; Deis, L. N.; Verma, V.; Keedy, D. A.; Hintze, B. J.; Chen, V. B.; Jain, S.; Lewis, S. M.; Arendall, W. B.; Snoeyink, J.; Adams, P. D.; Lovell, S. C.; Richardson, J. S.; Richardson, D. C. MolProbity: More and Better Reference Data for Improved All-Atom Structure Validation. *Protein Sci.* **2018**, *27* (1), 293–315.

(15) Lovell, S. C.; Davis, I. W.; Arendall, W. B.; De Bakker, P. I. W.; Word, J. M.; Prisant, M. G.; Richardson, J. S.; Richardson, D. C. Structure Validation by Cα Geometry: φ,ψ and Cβ Deviation. *Proteins Struct. Funct. Genet.* **2003**, *50* (3), 437–450.

(16) Chen, V. B.; Arendall, W. B.; Headd, J. J.; Keedy, D. A.; Immormino, R. M.; Kapral, G. J.; Murray, L. W.; Richardson, J. S.; Richardson, D. C. MolProbity: All-Atom Structure Validation for Macromolecular Crystallography. *Acta Crystallogr. Sect. D Biol. Crystallogr.* **2010**, *66* (1), 12–21.

(17) Yan, S.; Guo, C.; Hou, G.; Zhang, H.; Lu, X.; Williams, J. C.; Polenova, T. Atomic-Resolution Structure of the CAP-Gly Domain of Dynactin on Polymeric Microtubules Determined by Magic Angle Spinning NMR Spectroscopy. *Proc. Natl. Acad. Sci. U. S. A.* **2015**, *112* (47), 14611–14616.

(18) Loquet, A.; Bardiaux, B.; Gardiennet, C.; Blanchet, C.; Baldus, M.; Nilges, M.; Malliavin, T.; Böckmann, A. 3D Structure Determination of the Crh Protein from Highly Ambiguous Solid-State NMR Restraints. *J. Am. Chem. Soc.* **2008**, *130* (11), 3579–3589.

(19) Nieuwkoop, A. J.; Wylie, B. J.; Franks, W. T.; Shah, G. J.; Rienstra, C. M. Atomic Resolution Protein Structure Determination by Three-Dimensional Transferred Echo Double Resonance Solid-State Nuclear Magnetic Resonance Spectroscopy. *J. Chem. Phys.* **2009**, *131* (9).
